# Liquid Systems Based on Tetra(*n*-butyl)phosphonium Acetate for the Non-dissolving Pretreatment of a Microcrystalline Cellulose (Avicel PH-101)

**DOI:** 10.1021/acs.biomac.1c01683

**Published:** 2022-04-26

**Authors:** Carlos A. Pena, Alberto V. Puga, Andreas Metlen, Ana Soto, Héctor Rodríguez

**Affiliations:** † CRETUS, Department of Chemical Engineering, Universidade de Santiago de Compostela, E-15782 Santiago de Compostela, Spain; ‡ Departament d’Enginyeria Química, Universitat Rovira i Virgili, Avinguda dels Països Catalans 26, 43007 Tarragona, Spain; § AMT1—Translations & Chemistry, Schuurblok 11, 2910 Essen, Antwerp, Belgium

## Abstract

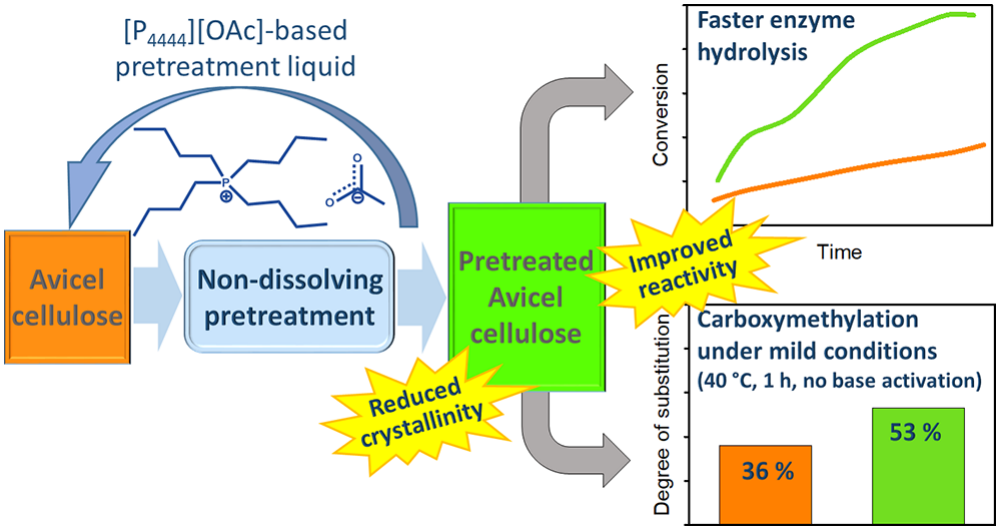

A non-dissolving pretreatment consisting in the direct contact of cellulose and the ionic liquid tetra(*n*-butyl)phosphonium acetate, or its fluid mixtures with other phosphonium ionic liquids or with molecular liquids such as ethanol or DMSO, causes a reduction in the crystallinity of the popular microcrystalline cellulose-type Avicel PH-101 under mild conditions. At the same time, the degree of polymerization and the thermal stability of the pretreated Avicel remain essentially unaltered with respect to the untreated Avicel. The diminution of the crystallinity has been related to the increase of the reactivity of the pretreated Avicel samples via analysis of the kinetics of their enzymatic hydrolysis. For selected samples, this improved reactivity has been confirmed through their effective carboxymethylation under a simplified and milder reaction procedure.

## Introduction

Within the general context of the Sustainable Development Goals set by the United Nations for 2030, the current industrial platform for the production of chemicals and materials is experiencing an intense shift toward the use of resources of biorenewable origin. Cellulose, which is the most abundant biopolymer on Earth, is likely to play a key role in that transition.^1^ This biopolymer is estimated to be produced by Nature at a rate higher than the capacity of the overall chemical industry worldwide, is not toxic, and has a very good geodistribution (especially if compared to the non-renewable resources that are currently fueling the global chemical industry).^2^ To a large extent, it is industrially obtained from (woody) lignocellulosic feedstock via a pulping process (e.g., Kraft, sulfite...). This pulping step barely affects the high degree of crystallinity of the cellulose naturally present in the lignocellulosic source. While this rigid and highly ordered molecular structure provides desirable mechanical properties for a number of applications (e.g., paper, packaging...), it also leads to a poorer reactivity and greater difficulty for the chemical transformation of cellulose into a large variety of added-value products (including cellulose-derived polymeric materials and non-polymeric chemicals such as cellulosic bioethanol, 5-hydroxymethylfurfural, and so forth).^3^ If the latter perspective is targeted, cellulose is typically subjected to a pretreatment stage for the improvement of its reactivity. This pretreatment constitutes a sustainability bottleneck in the overall cellulose processing since it often involves harsh temperature and/or pressure conditions along with the use of volatile organic solvents or strong acids or bases of poor green credentials.^1,4,5^

Ionic liquids are salts with a low melting or glass transition temperature (an arbitrary mark of 100 °C is typically considered).^6^ The properties of the compounds meeting this simple definition vary broadly. Nevertheless, many ionic liquids usually present a set of favorable attributes for their use as alternative solvents in potentially more sustainable processes: negligible vapor pressure (thus not contributing to air pollution or to the generation of flammable atmospheres), reasonably good thermal stability, and great solvation ability.^6,7^ In this context, some ionic liquids were found to dissolve cellulose (without derivatization) in relevant amounts under mild conditions,^8,9^ opening a door to their consideration as the basis of new cellulose processing technology. A good example of such a technology for a large market is the Ioncell-F process for the production of regenerated cellulose fibers, which avoids the use of the very toxic carbon disulfide necessary in the state-of-the-art Viscose process.^10,11^ Except in the case of direct transformation of cellulose into the desired products in the same ionic liquid dissolving medium, this dissolution strategy requires a complementary stage of regeneration of the dissolved cellulose. Due to the non-volatile character of both ionic liquid and cellulose, an antisolvent strategy (the addition of a substance miscible with the ionic liquid and with no capacity to dissolve cellulose) has been adopted in the scientific literature for such regeneration at a laboratory level.^12,13^ However, the large amounts of antisolvent needed (with water being by far the most popular antisolvent choice) are likely to lead to a prohibitive energy penalty in the vaporization of this antisolvent for recycling of the ionic liquid to the process at the industrial scale.^14^

Besides pretreatment methods based on the dissolution of cellulose with subsequent regeneration via a precipitation mechanism, a second alternative consists of a non-dissolving approach that typically uses volatile organic solvents or aqueous NaOH solutions (mercerization).^4,13,15^ Interestingly, the ability of ionic liquids to interact with cellulose is not restricted to those capable of dissolving it in relevant amounts. Some ionic liquids with negligible cellulose dissolution capacity have been proven to establish an effective interaction with the crystalline structure of cellulose.^16^ This enables the possibility of combining the potentially favorable attributes of ionic liquids (e.g., negligible vapor pressure, non-flammability, and avoidance of extreme pH conditions) with a mercerization-like approach, in which the large energy penalty brought about by the evaporation of the antisolvent (see the paragraph above) would be avoided. A promising ionic liquid for this purpose is tetra(*n*-butyl)phosphonium acetate ([P_4 4 4 4_][OAc]).^17,18^ Via an adequately integrated synthetic route, it has the potential to be produced industrially at a competitive cost.^19,20^ In addition, it presents good thermal stability^17^ and low toxicity,^21,22^ which are relevant aspects for its utilization in processes implemented at the industrial level. In a previous work, we have shown that, while having a negligible cellulose dissolution capacity, it is able to induce some decrystallization in microcrystalline cellulose.^17^

One of the disadvantages of [P_4 4 4 4_][OAc] is its relatively high melting temperature: 58 °C,^23^ requiring rather high temperatures for its application as a neat solvent.^18^ For its utilization in a pretreatment stage at a lower temperature, an option is to combine it with a second substance so that the melting temperature of the resulting mixture is lower. In that vein, we have recently reported that the combination of [P_4 4 4 4_][OAc] with homologous halides, namely, the organic salts tetra(*n*-butyl)phosphonium chloride ([P_4 4 4 4_]Cl) or tetra(*n*-butyl)phosphonium bromide ([P_4 4 4 4_]Br), produces eutectic systems with the melting temperature of the eutectic point being close to ambient temperature.^23^ This combination with a second ionic substance integrally preserves the advantageous non-volatile character. A different alternative is the combination with a molecular solvent, which instead will help to reduce not only the melting temperature but also, and considerably, the viscosity of the pretreatment fluid. Molecular co-solvents of interest may be ethanol or dimethylsulfoxide (DMSO), which can be taken as industrially used representatives of polar protic and polar aprotic solvents, respectively, and have reasonably good green credentials within their respective categories.^24^ Unfortunately, there is no previous knowledge of the solid–liquid equilibrium behavior of their mixtures with [P_4 4 4 4_][OAc] to guarantee beforehand the temperature range at which the corresponding pretreatments may take place.

In this work, different strategies are analyzed for the improvement of the technology based on [P_4 4 4 4_][OAc] for the pretreatment of cellulose. In particular, besides [P_4 4 4 4_][OAc] alone, the utilization of fluid mixtures of this ionic liquid with another [P_4 4 4 4_]-based ionic liquid or a molecular solvent, such as ethanol or DMSO, will be studied for the non-dissolving pretreatment of the widely used commercial microcrystalline cellulose type branded as Avicel PH-101 (hereinafter referred to as just “Avicel”, for simplicity).^25^ The effectiveness of the pretreatment on improving the reactivity of cellulose will be initially assessed in a simple way via analysis of the kinetics of its enzymatic hydrolysis, and it will be later evaluated with an application-oriented perspective in the reaction for the formation of a classical cellulose-modified product such as carboxymethylcellulose (CMC). The effect of temperature and composition will be explored in the Avicel pretreatment with these fluid mixtures. Along with the thermal stability and degree of polymerization (*DP*), the crystallinity index (*CI*) of the pretreated samples will be determined, and an attempt to relate it to the improvement observed in their reactivity will be carried out.

## Materials and Methods

### Materials

Avicel PH-101 (Sigma-Aldrich) was dried in an oven at 110 °C for ca. 2 days, to adjust its water content to ca. 1.5%, as determined from the weight loss of a thermogravimetric analysis (TGA) consisting on a heating ramp at 20 °C/min from room temperature to 110 °C, followed by a 15 min isotherm. This analysis was performed in a TA Instruments TGA Q500 thermogravimetric analyzer using N_2_ flow rates of 40 mL/min and 60 mL/min as balance purge gas and sample purge gas, respectively.

Ethanol (Panreac, 99.8%) and DMSO (Sigma-Aldrich, 99.99%) were used as received.

[P_4 4 4 4_]Cl was supplied by Iolitec with nominal purity >95%. [P_4 4 4 4_]Br was obtained from Sigma-Aldrich with a nominal purity of 98%. [P_4 4 4 4_][OAc] was produced in-house by the metathesis of [P_4 4 4 4_]Cl and potassium acetate (Sigma-Aldrich, 99%) according to a procedure described elsewhere.^17^ In brief, both reagents (K[OAc] in a 15% excess) were separately dissolved in ethanol and then mixed and stirred overnight in a beaker at room temperature. The precipitate was removed by vacuum filtration through a sintered funnel and the solvent by rotary evaporation. The residue (crude [P_4 4 4 4_][OAc]) was redissolved in acetone (Scharlau, ≥99.8%) and placed in a freezer for 24 h to promote further precipitation. Filtration, rotary evaporation, redissolution in fresh acetone, and placement in the freezer were repeated until no further precipitate was observed.

All three phosphonium salts were subjected to purification under reduced pressure (absolute pressure lower than 1 Pa) while being magnetically stirred and heated up to ca. 380 K ([P_4 4 4 4_]Cl and [P_4 4 4 4_]Br) or ca. 345 K ([P_4 4 4 4_][OAc]). The ^1^H and ^13^C NMR spectra of the purified products are shown in Figures S1–S6 in the Supporting Information, confirming the expected chemical identities of the compounds and the absence of organic impurities at relevant levels. For [P_4 4 4 4_][OAc], the residual Cl^–^ and K^+^ concentrations were 1000 ppm (determined by ion chromatography using a Metrohm 861 Advanced Compact IC chromatograph equipped with a Metrosep A Supp5 250/4.0 mm column, with an aqueous solution of 3.2 mM sodium carbonate and 1.0 mM sodium bicarbonate as the mobile phase) and <4 ppm (determined by ICP-OES using a PerkinElmer Optima 4300 DV spectrometer equipped with a 40 MHz RF plasma generator), respectively. The water content of the purified phosphonium salts was determined by the Karl-Fischer titration method in a Metrohm 899 coulometer, and water mass fractions lower than 300 ppm were found in all cases.

### Solid–Liquid Equilibria

Solid–liquid equilibrium behaviors of binary systems were determined by differential scanning calorimetry (DSC). For each system, samples with a step composition of ca. 0.10 in mole fraction, and covering the entire composition range, were prepared in small glass vials with the assistance of a Mettler-Toledo XPE205 analytical balance with an uncertainty of 1 × 10^–4^ g. After homogenization by thorough stirring, at ambient temperature or with mild heating, aliquots of 5–20 mg of each sample were transferred to 40 mL DSC aluminum pans (manufactured by TA Instruments) and sealed hermetically with the corresponding aluminum lids (also manufactured by TA Instruments). The sealed capsules were loaded into the measuring chamber of a TA Instruments Q2000 differential scanning calorimeter equipped with an RCS 90 refrigerated cooling system, and an analogous capsule with no sample was used as the empty reference. A flow rate of 50 mL/min of N_2_ (Nippon Gases, 99.999%) was applied as the purge gas. The DSC thermal program consisted of an initial heating ramp at 5 °C/min from ambient temperature to 80 °C, followed by two cycles, each of them comprising a cooling ramp at −5 °C/min down to −90 °C and a heating ramp at 5 °C/min up to 80 °C, with intercalated 10 min isotherms in between ramps. (Due to the loss of baseline stability at very low temperatures, the portion of thermograms below −70 °C was systematically disregarded.) It was verified that the signals of the two cycles were repetitive. The signal of the last heating ramp was used to evaluate the thermal events of the sample (glass transition temperatures at the midpoint of the sigmoidal portion of the thermogram resulting from the variation in heat capacity and melting temperatures at the onset of the corresponding endothermic peak) with the software Universal Analysis 2000, version 4.5.0.5 by TA Instruments. Uncertainty associated with the reported temperatures was estimated to be 1 °C.

### Characterization of the Pretreatment Fluids

Density and viscosity of the pretreatment fluids were simultaneously determined in an Anton Paar DMA 5000 M oscillating U-tube density meter with an Anton Paar LOVIS 2000 ME microviscometer module attached based on the rolling ball principle. The density meter chamber has two integrated Pt 100 platinum thermometers together with Peltier elements for the precise thermostating of the sample (temperature accuracy: 0.01 °C) and performs an automatic correction of viscosity-related errors over the full viscosity range. Uncertainty of the density values obtained was estimated to be 1 × 10^–5^ g/cm^3^. The capillary of the microviscometer module, located into a temperature-controlled block (temperature accuracy: 0.02 °C), was adjusted with a certified viscosity standard fluid by Anton Paar. The dynamic viscosity values thus obtained are accurate to within 0.5%.

### Pretreatment of Avicel

In each pretreatment experiment, 10.0 g of the selected pretreatment fluid was placed in a jacketed glass cell, and 1.00 g of Avicel was added (thus corresponding to a solid-to-liquid ratio of 10 g of solid per 100 g of liquid). The glass cell was stoppered, and water from a Selecta Ultraterm 200 thermostatic bath was circulated through the jacket to keep the mixture at the desired temperature (with an uncertainty of 0.1 °C). The solid–liquid contact was promoted via mechanical stirring (ca. 150 rpm) using an IKA RW 16 Basic overhead stirrer. After 2 h, the stirring was ceased, and the content of the cell was filtered under soft vacuum using a fritted glass Allihn filter tube coupled with a Kitasato flask. The recovered solid was washed with 50 mL portions of bidistilled water until a residual concentration of ionic liquid lower than 50 ppm in the washing waters was obtained. Such a concentration was ascertained, through the corresponding calibration curve (see Tables S1–S3 and Figures S7–S9 in the Supporting Information), by UV absorbance at 195 nm in an Agilent 8453 UV–vis spectrophotometer equipped with a deuterium plasma discharge lamp. In the particular cases of utilization of a mixture of [P_4 4 4 4_][OAc] and DMSO as the pretreatment fluid, the absence of DMSO in the pretreated Avicel was also checked by the analysis of the total sulfur content of the washing waters in an Oxford Instruments Lab-X3500S spectrometer (detection threshold: 2.5 ppm). Once sufficiently washed, the pretreated Avicel sample was placed in a mortar and dried in an oven at 110 °C, with periodical soft grinding with the help of a pestle to avoid agglomeration of particles, until obtaining a water content similar to that of the non-pretreated Avicel (as determined by TGA—see subsection Materials).

For verification of the non-dissolving character of the studied pretreatments, 2.00 g of the pretreatment fluid was placed into the glass cells and thermostated at the specified pretreatment temperatures, and ca. 0.01 g of Avicel was added, thus yielding a ratio of 0.5 g of cellulose per 100 g of liquid. After stirring the heterogeneous mixture magnetically (150 rpm) for 24 h, a sample was taken for analysis by confocal microscopy in a Leica TCS SP5 X microscope using a UV 405 nm diode and a Scan-DIC-Pol filter.

### Characterization of Raw and Pretreated Avicel Samples

The *CI* of Avicel samples was determined using powder X-ray diffractometry (PXRD), using a Bruker D8-Advance diffractometer with Cu Kα radiation (λ = 1.5406 Å), equipped with a LYNXEYE-2 detector and a rotary sample holder for Bragg–Brentano geometry. The X-rays were produced in a Cu-sealed tube, and the radiation was monochromatized with a graphite monochromator (λ(Kα1) = 1.5406 Å). The diffractograms were recorded in an angular range 13–30°, with a step of 0.02° and an accumulation time of 6 s. The samples were rotated during the analysis in order to get the optimal peak profiles as well as to minimize the effect of preferential orientation. Subtraction of the amorphous content, currently considered as the strategy to lead to numerically most reliable results,^25,26^ was carried out thanks to the parallel analysis of an Avicel sample totally amorphized. To obtain the latter, a sample of Avicel was introduced together with a zirconia ball of 2 cm of diameter in the 20 cm^3^ zirconia chamber of a Retsch MM-2 vibration mill, leaving more than 50% of free space in the chamber for the milling to be performed correctly (according to the specifications by the manufacturer). This ball-milling was carried out for 30 min with a vibration frequency of 900 cycles/min. The *CI* was calculated as the difference of the areas under the curves of the specific Avicel sample and the one totally amorphized, after a process of normalization of the curves. Each reported diffractogram is the average of two independent replicates.

The *DP* was determined via a procedure based on the measurement of the intrinsic viscosity.^27^ Initially, 0.130 g of the Avicel sample was dissolved in 5.0 mL of a 0.5 M solution of bis(ethylenediamine)copper(II) hydroxide (supplied by Sigma-Aldrich as an aqueous solution of concentration 1.0 M) under an inert atmosphere of argon (Praxair, ≥99.9%). The kinematic viscosity (ν) of this solution was measured with a micro-Ubbelohde glass capillary viscometer (manufactured, calibrated, and certified by Schott) at 25 °C, using an automatic Lauda PVS1 Process Viscosity System equipped with a Lauda D20 KP clear-view water bath thermostat coupled with a Lauda DLK 10 through-flow cooler for temperature control (temperature uncertainty: 0.05 K), and with a photoelectric cell for the precise determination of the efflux time of the liquid sample through the capillary (time resolution: 0.01 s). The quotient of this kinematic viscosity over the kinetic viscosity of the 0.5 M aqueous solution of bis(ethylenediamine)copper(II) hydroxide (without any cellulose dissolved) is the dynamic relative viscosity, which can be related through literature tables with the product of the intrinsic viscosity ([η]_*c*_).^27^ Then, the *DP* can be obtained by means of the following expression:
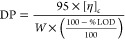
1where *W* is the mass (in grams) of the cellulose dissolved in the 0.5 M aqueous solution of bis(ethylenediamine)copper(II) hydroxide and %LOD is the percentage of weight loss observed by TGA of the cellulose sample after having been subjected to the drying process in the oven, as described in subsection Materials.

### Kinetics of the Enzymatic Hydrolysis of Avicel Samples

Citric acid (Sigma-Aldrich, 99.5%) and sodium citrate (Sigma-Aldrich, 99%) were used to prepare an aqueous 0.01 M citrate buffer (pH = 5). In each hydrolysis experiment, 10 mL of this buffer solution was placed in a jacketed glass cell, along with 0.10 g of the corresponding Avicel sample. The content of the cell was magnetically stirred (150 rpm) and kept at 50 °C by means of circulating water from a thermostatic water bath. A volume of 30 μL of a 1:3 dilution of the commercial enzyme blend Cellic CTec2 by Novozymes (containing cellulases, β-glucosidases, and hemicellulase) was added to initiate the hydrolysis. Aliquots of the liquid were taken at different times as the hydrolysis progressed, and their glucose concentration was analyzed with a commercial enzymatic kit by Spinreact. This kit is based on the oxidation of glucose to gluconic acid by glucose oxidase, producing an equimolar amount of hydrogen peroxide. The latter is then detected by a chromogenic oxygen acceptor (phenol + 4-aminophenazone) in the presence of peroxidase, yielding an intense color that can be quantified by the measurement of the absorbance at 505 nm. Within the adequate range, this absorbance is proportional to the glucose concentration in the sample.^28,29^ Samples were filtered through 0.45 μm pore size disposable filters and diluted with a 9 g/L solution of sodium chloride (Sigma-Aldrich, 99.5%). Subsequently, they were incubated for 30 min (in a thermostated Selecta Boxcult orbital shaker) at 25 °C, with a stirring angular velocity of 150 rpm. The absorbance was measured in an Agilent 8453 UV–vis spectrophotometer using a quartz cuvette with a 10 mm light path. A standard glucose solution of 1 g/L was used to determine the absorbance correction factor. All determinations of glucose concentration were carried out in triplicate, typically with a repeatability of 3–4%, and the average values were reported.

### Carboxymethylation of Avicel Samples

In a typical carboxymethylation experiment, 0.100 g of the Avicel sample and 0.120 g of sodium chloroacetate (Sigma-Aldrich, for synthesis) were weighed in a 10 mL glass tube and suspended in 2.0 mL of 2-propanol (Scharlau, ≥99.5%). The tube was then immersed in an oil bath preheated at 40 °C, and the suspension was magnetically stirred at 1000 rpm for 2 min. Then, 0.1 mL of a ca. 20% w/v aqueous solution of sodium hydroxide (Sigma-Aldrich, ≥98%), corresponding to an equimolar [OH]^−^/anhydroglucose unit ratio, was added. The suspension was stirred at 1000 rpm for the desired time (1 h or 3 h), and after this reaction period, the tube was taken out of the bath and allowed to settle and cool down to room temperature, and its content was centrifuged at 2000 rpm for 3 min. The supernatant was carefully decanted using a pipette, and the sedimented solid was washed with 2-propanol (3 × 1 mL) by stirring at room temperature for 2 min and then centrifuging-decanting as described above. The resulting solid was suspended in 6 mL of methanol (Merck, ≥99.9%), and the stirred mixture was neutralized until pH ≈ 6 (as measured using pH indicator paper) by adding a few drops of glacial acetic acid (Scharlau, extrapure). The solid was separated by filtration on a sintered glass funnel (pore size: 3), washed with methanol (5 × 2 mL), and dried under an air stream by suction. The final CMC samples had a white color, and their texture ranged from powdery solids to slightly sticky flakes depending on their degrees of substitution.

The degree of substitution (*DS*) of the CMCs was determined by attenuated total reflectance Fourier-transform infrared (ATR-FTIR) spectroscopy, suitable for *DS* determination with a small amount of sample (<10 mg). After recording the spectra on a Jasco FT/IR instrument, *DS* values were estimated from the absorbance ratio (*R*_CM_) between the signals for the asymmetric carboxylic stretching and the methylenic/methinic stretching, designated as ν_as_(COO^–^) and ν(CH), respectively. Such a ratio was calculated as *R*_CM_ = *I*_COO_/*I*_CH_, where *I*_COO_ and *I*_CH_ are the peak heights, after baseline correction, of the ν_as_(COO^–^) and ν(CH) signals found at ca. 1590 and 2900 cm^–1^, respectively. Calibration of this ATR-FTIR method was done by correlating *R*_CM_ to actual *DS* figures, determined by acid–base back-titration, for a series of CMC samples (see details in the Supporting Information, including Table S4 and Figures S10–S11).

## Results and Discussion

### Liquid Systems and Temperatures for Pretreatment

Pure [P_4 4 4 4_][OAc] has a reported melting temperature of 58 °C.^23^ For its use for the pretreatment of Avicel, a temperature of 70 °C was selected.^17^

To explore the possibility of carrying out a pretreatment with a similar effect, but at a lower temperature (with the associated savings in energy demand), the eutectic mixtures of [P_4 4 4 4_][OAc] with [P_4 4 4 4_]Cl and with [P_4 4 4 4_]Br were considered. These mixtures will preserve the intrinsic ionic liquid advantages of the pretreatment fluid while representing only a partial replacement of the acetate anions (key in the interaction of the ionic liquid with cellulose due to their basicity) with halides that also exhibit some basicity—they are Lewis bases. The eutectic point for the system [P_4 4 4 4_][OAc] + [P_4 4 4 4_]Cl corresponds to a nearly equimolar composition at 24 °C, whereas the one for the system [P_4 4 4 4_][OAc] + [P_4 4 4 4_]Br corresponds to an approximate composition of 70 mol % of [P_4 4 4 4_][OAc] at 32 °C.^23^ Therefore, it was decided to use these eutectic compositions as pretreatment fluids for Avicel at a temperature of 40 °C. For direct comparison with the performance of neat [P_4 4 4 4_][OAc], equivalent pretreatments at 70 °C were also included in the study. As a complementary characterization of these liquid systems intended for the pretreatment of Avicel cellulose at more than one temperature, two of their key properties from an industrial process design, such as density and viscosity, were measured as a function of temperature. The detailed numerical values of the measurements and their graphical evolution with temperature are presented in Table S5 and Figure S12 in the Supporting Information. The parameters of the corresponding correlations by means of linear/quadratic fits (for density) and the Vogel–Fulcher–Tammann equation (for viscosity) are shown in Table S6.

**Figure 1:**
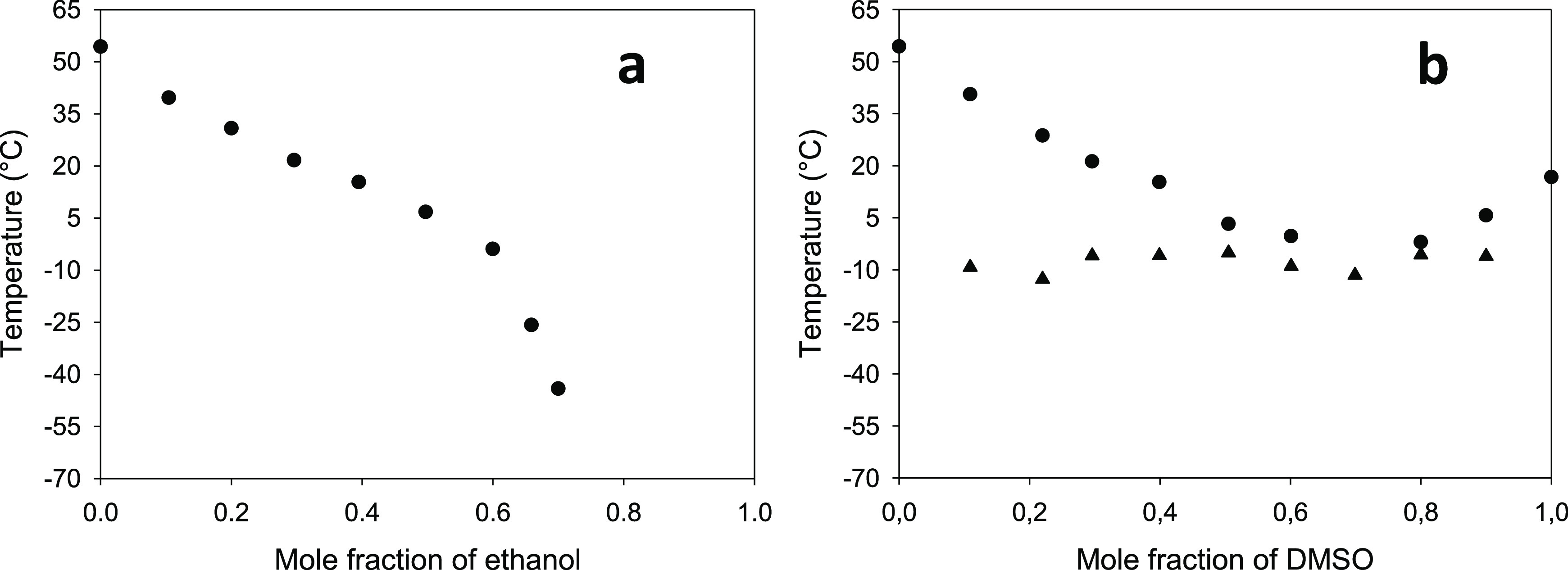
Temperature–composition diagrams for the solid–liquid phase equilibria of the binary systems [P_4 4 4 4_][OAc] + ethanol (plot a) and [P_4 4 4 4_][OAc] + DMSO (plot b—Legend: circle, excess component melting; triangle, eutectic melting). For samples with an ethanol mole fraction higher than 0.70, no thermal events were detected above ca. −70 °C (the lower limit of the reliable temperature range of the DSC instrument used).

An alternative for the pretreatment at lower temperatures is the use of mixtures of [P_4 4 4 4_][OAc] with organic solvents. These organic solvents will present the inconvenience of their volatility and the risks associated with it (flammability, atmospheric pollution, loss of solvent by evaporation, etc.). However, they will also lead to a number of advantages: lowering the viscosity of the pretreatment fluid, decreasing its cost per unit mass, and also the possibility of potentially reducing the melting temperature in a significant manner with respect to pure [P_4 4 4 4_][OAc]. In particular, ethanol and DMSO were selected in this study, representing a polar protic solvent and a polar aprotic solvent, respectively. Water was disregarded due to its very high hydrogen bond donor ability, which would invalidate the basicity of the anion necessary to interact with the cellulose substrate for disruption of its hydrogen-bonding network and would likely lead to an inefficient pretreatment.^17^ Two different concentrations of molecular cosolvent, namely, 0.20 and 0.40 in mole fraction, were selected in each case. For the rigorous choice of an appropriate pretreatment temperature, the solid–liquid equilibria of the binary systems [P_4 4 4 4_][OAc] + ethanol and [P_4 4 4 4_][OAc] + DMSO were experimentally investigated at atmospheric pressure. The corresponding temperature–composition diagrams are presented in Figure 1. The numerical values associated with the preparation of these diagrams are listed in Tables S7 and S8 in the Supporting Information. A sustained decrease in the melting temperature was observed in the system [P_4 4 4 4_][OAc] + ethanol with an increase in the concentration of ethanol, at least up to an ethanol mole fraction of 0.70. In the ethanol mole fraction 0.70–1.00, no solid–liquid transition was detected within the reliable experimental temperature range of the DSC instrument used. Regarding the system [P_4 4 4 4_][OAc] + DMSO, a clear eutectic behavior could be identified, with the eutectic point corresponding to a composition of ca. 0.70 in the mole fraction of DMSO and a temperature of −12 °C. By inspection of these diagrams, a temperature of 40 °C was initially selected for the low-temperature pretreatments since all mixtures [P_4 4 4 4_][OAc] + (ethanol or DMSO) with a cosolvent mole fraction of 0.20 and 0.40 are liquid at this temperature. However, preliminary tests for the pretreatment of Avicel showed a tremendous rise in viscosity at this low temperature in the [P_4 4 4 4_][OAc] + DMSO mixtures, which could be notably mitigated if operating at 50 °C. Therefore, this temperature of 50 °C was the final choice to uniformly perform the low-temperature pretreatments with the combinations of ionic liquid and molecular solvent. For direct comparison with pure [P_4 4 4 4_][OAc], analogous pretreatments at 70 °C were also performed. In a similar vein to what was done for the eutectics of the mixtures of phosphonium salts, the density and viscosity were also measured as a function of temperature for these additional solvent systems. The numerical values and their graphical representation are included in Table S5 and Figures S13 and S14 in the Supporting Information, while the parameters of the corresponding correlations describing these properties over the investigated temperature range are shown in Table S6.

### Pretreatment of Avicel

**Figure 2:**
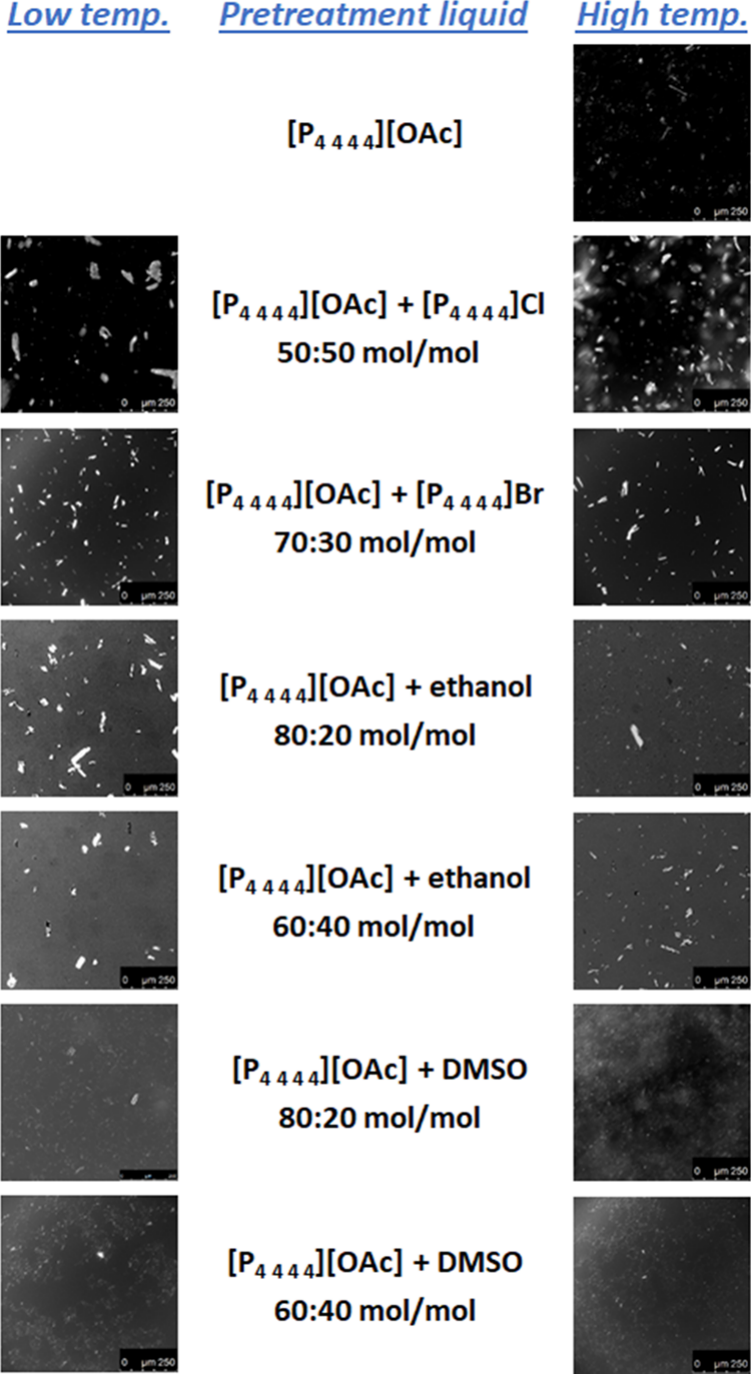
Microscopic images, at a magnification of 20×, of mixtures of Avicel and pretreatment liquid in the ratio 0.5:100 (w/w), after vigorous stirring for 24 h at constant temperature. Photographs in column *High temp.* correspond to pretreatment temperatures of 70 °C, whereas photographs in column *Low temp.* correspond to pretreatment temperatures of 40 °C for the eutectics of [P_4 4 4 4_][OAc] + [P_4 4 4 4_]Cl and [P_4 4 4 4_][OAc] or 50 °C for the mixtures of [P_4 4 4 4_][OAc] + (ethanol or DMSO).

Although it was already evident from direct visual observation, the non-dissolving character of the pretreatments to be investigated was assessed by optical microscopy. Figure 2 shows microscopic photographs of mixtures where Avicel and the pretreatment liquid were combined in a ratio of 0.5:100 (w/w) and stirred vigorously for 24 h at the corresponding pretreatment temperature. The presence of the undissolved material in all cases (although with evident reduction of the size of the observable particles in some cases) indicates that the solubility of Avicel in the tested pretreatment fluids is lower than 0.5 g of cellulose per 100 g of fluid. Thus, the assumption of non-dissolving pretreatment was validated.

**Figure 3:**
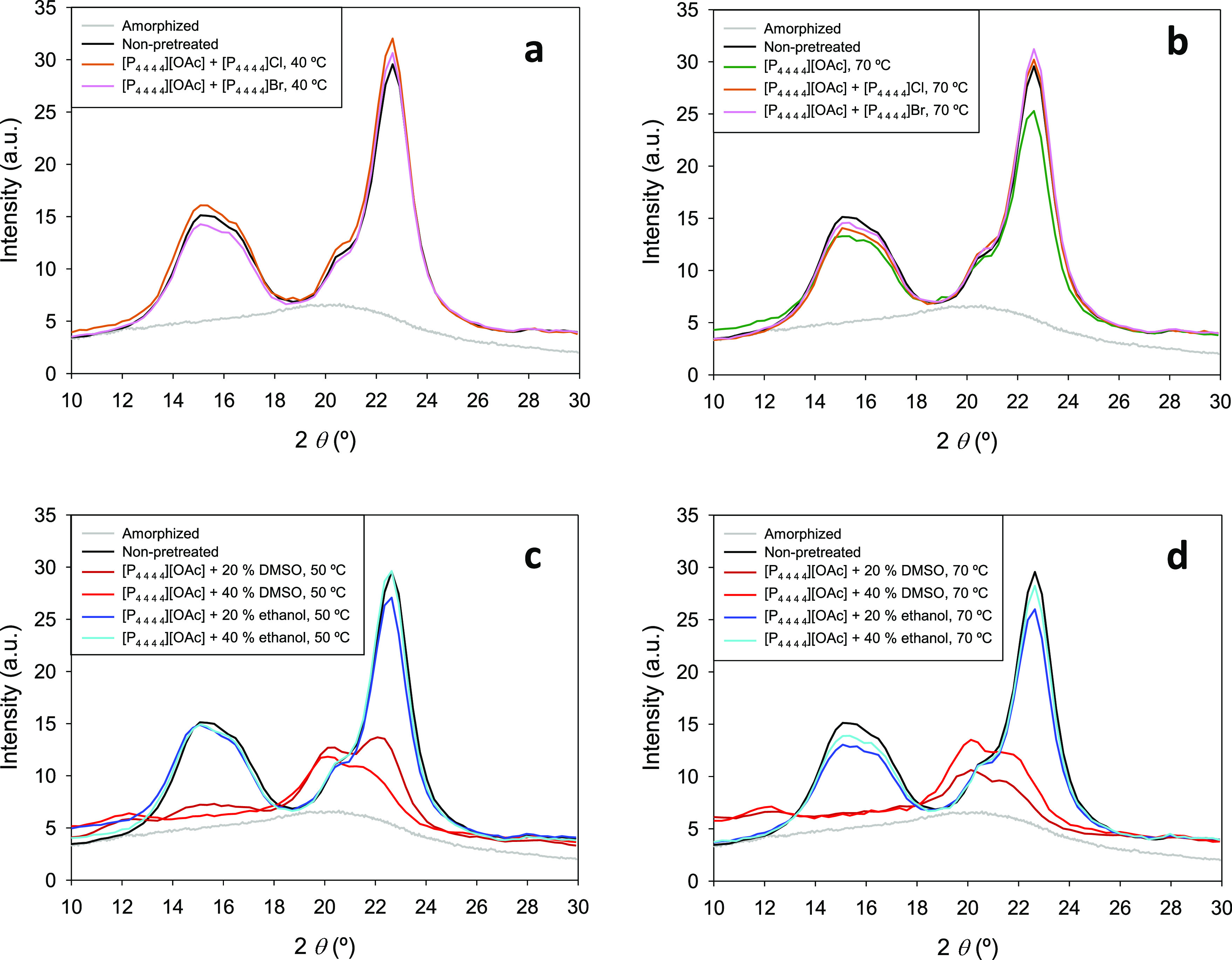
PXRD diffractograms of samples pretreated at 40 °C (plot a) or 70 °C (plot b) with pure [P_4 4 4 4_][OAc] (green line) or its eutectic mixtures with [P_4 4 4 4_]Cl (orange line) and [P_4 4 4 4_]Br (pink line) and at 50 °C (plot c) or 70 °C (plot d) with mixtures of [P_4 4 4 4_][OAc] + ethanol with *x*_ethanol_ = 0.20 (dark blue line) or *x*_ethanol_ = 0.40 (light blue line) or with mixtures of [P_4 4 4 4_][OAc] + DMSO with *x*_DMSO_ = 0.20 (dark red line) or *x*_DMSO_ = 0.40 (light red line). The diffractogram of non-pretreated Avicel cellulose (black line) is also shown in all plots as the reference, as well as that of the amorphized Avicel (gray line).

The PXRD diffractograms of the Avicel samples pretreated with the different liquid systems at the selected temperatures are shown in Figure 3, where the diffractogram of untreated Avicel is also shown for comparison. For the Avicel sample pretreated with neat [P_4 4 4 4_][OAc] (at 70 °C), a decrease in the intensity of the crystalline peaks is observed with respect to the Avicel sample with no pretreatment. The associated *CI* values are listed in Table 1, indicating a decrease from 51% for the untreated sample to 46% for the pretreated Avicel.

**Table 1:** Density (ρ) and Dynamic Viscosity (η) of the Pretreatment Liquids, CI, DP, and 5% Onset Decomposition Temperature (*T*_d,5%onset_) for the Samples of Avicel Pretreated with the Indicated Liquids at the Corresponding Temperatures *T*

pretreatment fluid	*T* (°C)	ρ (g/cm^3^)	η (mPa·s)	*CI* (%)	*DP*	*T*_d,5%onset_ (°C)
no pretreatment				51	221	291
[P_4 4 4 4_][OAc]	70	0.90886	43.90	46	204	293
[P_4 4 4 4_][OAc] + [P_4 4 4 4_]Cl, 50:50 mol/mol	40	0.93018	799.4	52	210	294
	70	0.91315	121.1	50	212	287
[P_4 4 4 4_][OAc] + [P_4 4 4 4_]Br, 70:30 mol/mol	40	0.96358	412.5	51	210	295
	70	0.94583	85.20	51	211	297
[P_4 4 4 4_][OAc] + ethanol, *x*_ethanol_ = 0.20	50	0.91527	77.94	44	210	267
	70	0.90338	32.38	46	209	270
[P_4 4 4 4_][OAc] + ethanol, *x*_ethanol_ = 0.40	50	0.90707	40.57	45	213	274
	70	0.89494	20.63	48	212	289
[P_4 4 4 4_][OAc] + DMSO, *x*_DMSO_ = 0.20	50	0.92755	71.24	29	207	268
	70	0.91559	29.87	20	202	271
[P_4 4 4 4_][OAc] + DMSO, *x*_DMSO_ = 0.40	50	0.93768	42.35	23	210	267
	70	0.92531	19.49	28	211	275

Regarding the eutectic mixtures of phosphonium salts, none of their pretreatments (either at 40 °C or at 70 °C) caused a significant variation of the crystallinity with respect to the untreated Avicel, as evidenced in Figure 3a,b, and also numerically by the corresponding *CI* values in Table 1. Besides the reduction in the intensity of the basicity caused by the partial replacement of acetate anions with halide anions (and therefore the reduction in the ability to interact with the hydroxyl groups of cellulose), another possible contributing factor to this lack of crystallinity reduction in the pretreatment could be the much higher viscosity of the eutectic mixtures at the investigated temperatures, as compared to the viscosity of pure [P_4 4 4 4_][OAc]—see Table 1. Such a viscosity might hamper the necessary mobility for interaction between the ions of the pretreatment fluid and the cellulose structures.

The PXRD diffractograms of the Avicel samples pretreated with the mixtures [P_4 4 4 4_][OAc] + (ethanol or DMSO) can be seen in Figure 3c,d. The diffractograms from the pretreatments with [P_4 4 4 4_][OAc] + ethanol respond to the same pattern of those pretreatments with integrally ionic fluids. The degree of crystallinity reduction achieved in these cases is similar to that obtained in the pretreatment with neat [P_4 4 4 4_][OAc], with the corresponding *CI* values listed in Table 1 showing a slightly larger decrystallization effect in the pretreatments at 50 °C. Regarding the [P_4 4 4 4_][OAc] + DMSO mixtures, a strong reduction of the areas under the signals can be observed, evidencing a remarkable decrease in the cellulose crystallinity. Moreover, a shift in the peaks is produced, from signals characteristic of the native Cellulose I crystalline structure (e.g., at 22.5°) to signals corresponding to the more thermodynamically stable Cellulose II crystalline structure (e.g., the region 20–21.5°).^30^ Despite the evidence of this allomorphic transformation, solubility tests revealed a solubility lower than 0.5 g per 100 g of pretreatment fluid for either of the investigated mixtures of [P_4 4 4 4_][OAc] and DMSO. The optical microscopic photographs for such solubility tests in Figure 2 show undissolved particles after 24 h at the pretreatment temperature; however, it is clear that these particles are less and smaller in the mixtures with DMSO than in the other liquid systems tested. Therefore, it may be possible that the transformation of Cellulose I to Cellulose II exists as the result of a dynamic equilibrium of solubilization of cellulose in the [P_4 4 4 4_][OAc] + DMSO pretreatment fluids, although keeping the overall solubility at levels that preserve the general non-dissolving character of the pretreatment at the macroscopic scale.

The special performance of the [P_4 4 4 4_][OAc] + DMSO in reducing the crystallinity of the cellulose sample should be attributed to the capacity of the polar aprotic solvent to disrupt the ionic association of [P_4 4 4 4_][OAc], thereby releasing more free ions to interact with cellulose molecules (mainly through hydrogen bonding) through the solvation of cations and anions, without penalizing the ability of these ions to interact with cellulose, mainly through hydrogen bonding.^31^ The known cellulose swelling ability of DMSO may enable an initial cellulose swelling step, enhancing the accessibility of [P_4 4 4 4_][OAc] to cellulose and therefore facilitating the subsequent decrystallization and allomorphic transformation,^31^ in a similar fashion to what has been reported for the dissolution of cellulose in mixtures of tetra(*n*-butyl)ammonium acetate and DMSO.^32,33^

The strong reduction of the crystallinity of Avicel in the pretreatment with the [P_4 4 4 4_][OAc] + DMSO mixtures is obviously reflected in the remarkably lower *CI* values displayed in Table 1 for these cases. Interestingly, a different trend of *CI* with the pretreatment temperature is observed for the mixture with *x*_DMSO_ = 0.20 (*CI* decreases from 29% for the pretreatment at 50 °C to 20% for the pretreatment at 70 °C) and the mixture with *x*_DMSO_ = 0.40 (*CI* increases from 23% for the pretreatment at 50 °C to 28% for the pretreatment at 70 °C). These opposed trends are likely the result of an equilibrium between two factors: the interaction forces (mainly hydrogen bonding) of the pretreatment liquid with the cellulose chains to modify their 3D structure, which weaken with an increase in temperature, and the fluidicity (i.e., the inverse of the viscosity) of the pretreatment fluid, which facilitates the mass transfer stages in the mechanism of decrystallization and increases with increasing temperature. As observed in Table 1, the viscosities of the [P_4 4 4 4_][OAc] + DMSO mixtures at 50 °C are practically double than their values at 70 °C. In the case of the mixture with a composition *x*_DMSO_ = 0.20, the increased transport barrier is likely having a stronger overall effect on the pretreatment process than the enhancement of the hydrogen-bonding interaction as a result of the lower temperature, with the contrary applying to the mixture with composition *x*_DMSO_ = 0.40.

The preservation of the length of the polymeric chains and of the thermal stability are aspects of interest for a number of cellulose applications. The corresponding values of *DP* and *T*_d,5%onset_ (used herein as a numerical indicator of the thermal stability) for the Avicel samples resulting from all the above pretreatments are listed in Table 1. Despite the important *CI* variations commented throughout this section, the described pretreatments led to only a small *DP* reduction of ca. 5–10% with respect to the raw Avicel. Regarding the thermal stability, all the *T*_d,5%onset_ values of the pretreatments with integrally ionic fluids lie within ±2% of the value of the untreated Avicel, thus implying a negligible effect on the thermal stability of the cellulosic material. For the pretreatments with mixtures of [P_4 4 4 4_][OAc] and a molecular solvent, a small decrease in *T*_d,5%onset_ was systematically observed, never larger than ca. 8% (the lowest *T*_d,5%onset_ value being 267 °C), and therefore, it can still be considered that, for most practical purposes, the thermal stability of the pretreated Avicel is well preserved.

### Enzymatic Hydrolysis

**Figure 4:**
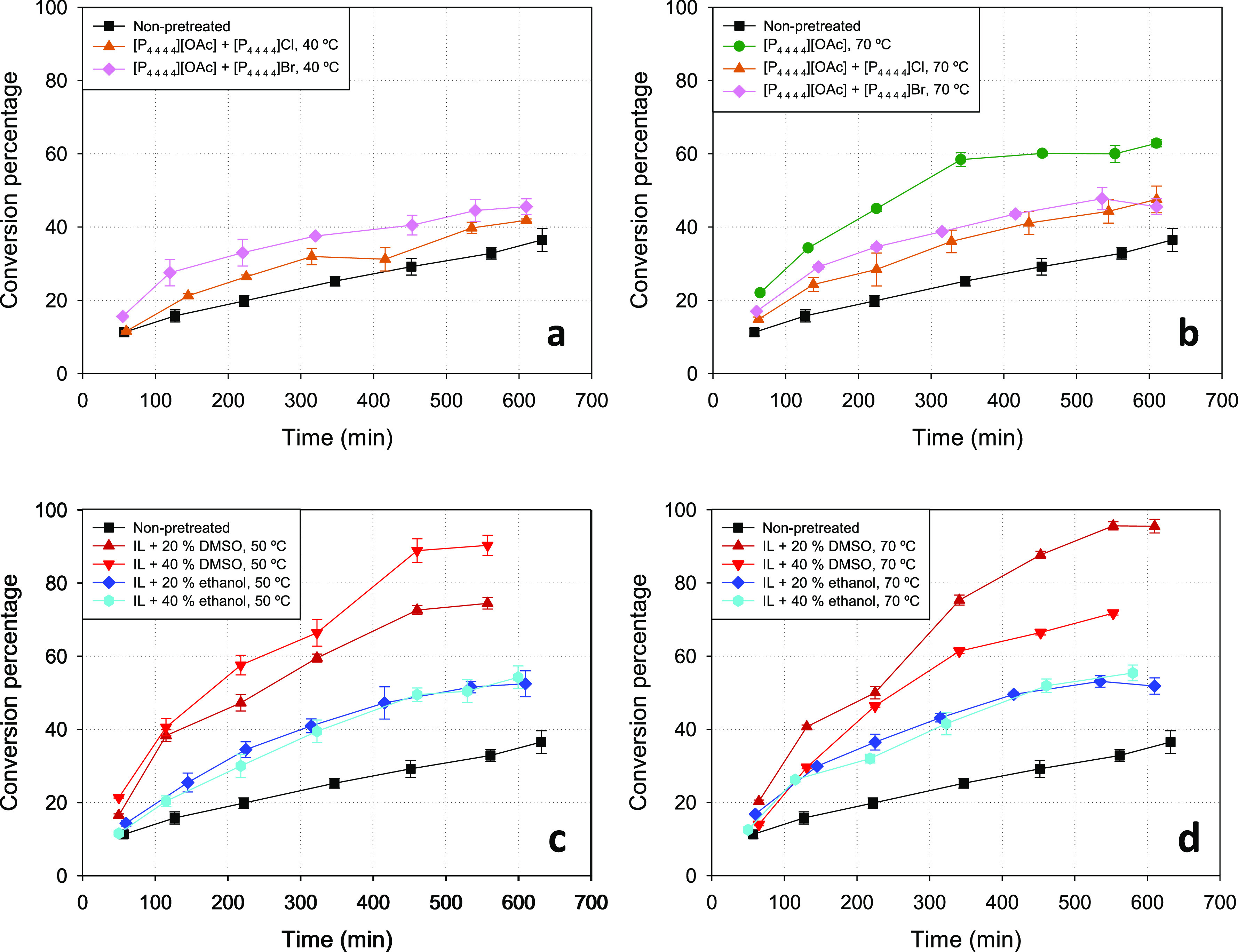
Evolution with time of the percentage of conversion of cellulose into glucose in the reaction of enzymatic hydrolysis of the Avicel samples pretreated at 40 °C (plot a) or 70 °C (plot b) with pure [P_4 4 4 4_][OAc] (green circles) or its eutectic mixtures with [P_4 4 4 4_]Cl (orange triangles) and [P_4 4 4 4_]Br (pink diamonds) and at 50 °C (plot c) or 70 °C (plot d) with mixtures of [P_4 4 4 4_][OAc] (IL) + ethanol with *x*_ethanol_ = 0.20 (dark blue diamonds) or *x*_ethanol_ = 0.40 (light blue hexagons) or with mixtures of [P_4 4 4 4_][OAc] (IL) + DMSO with *x*_DMSO_ = 0.20 (dark red triangles) or *x*_DMSO_ = 0.40 (light red inverted triangles). The corresponding curve for the hydrolysis of untreated Avicel (black squares) is also shown in all plots as the reference. Lines are a guide to the eye.

**Figure 5:**
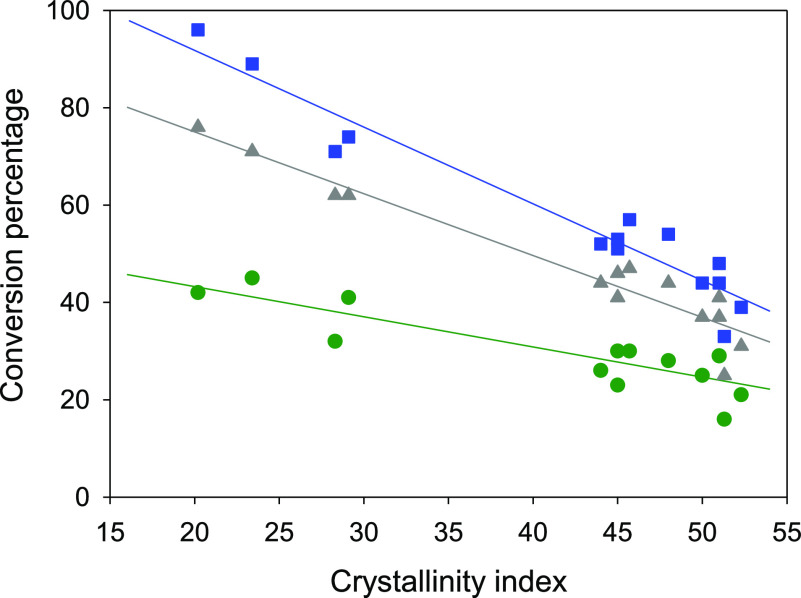
Extension of the enzymatic hydrolysis of Avicel cellulose samples as a function of their *CI* for selected reaction times: 150 min (green, circles), 350 min (gray, triangles), and 550 min (blue, squares). Conversion values at the specified times were obtained as linear interpolations in the respective hydrolysis time courses. Lines correspond to the best linear fit for each series.

The kinetic curves of the enzymatic hydrolysis of the pretreated Avicel samples, as well as of untreated Avicel, are shown in Figure 4. All hydrolyses of the pretreated samples occurred faster than that of untreated Avicel, thus indicating an improvement in the reactivity conferred by the pretreatment stage. The fastest hydrolyses corresponded to the Avicel samples pretreated with [P_4 4 4 4_][OAc] + DMSO mixtures, for which the largest reduction in crystallinity was observed (see subsection Pretreatment of Avicel). This is in line with what was observed in our previous work with microcrystalline cellulose.^17^ Interestingly, there is a good correspondence of the *CI* values obtained for the Avicel samples pretreated with these [P_4 4 4 4_][OAc] + DMSO mixtures (see Table 1) and their rate of enzymatic hydrolysis: the fastest hydrolysis of the samples pretreated at 50 °C corresponds to pretreatment with the mixture with *x*_DMSO_ = 0.40, whereas for the samples pretreated at 70 °C, it corresponds to pretreatment with the mixture with *x*_DMSO_ = 0.20 (Figure 4, plots c and d). Expanding the exploration of a relation between the *CI* of Avicel samples and their enzymatic hydrolysis kinetics to the entire set of samples investigated in this work, we produced the plot in Figure 5, where a reasonably linear decrease can be observed (albeit with substantial scattering in the region of high crystallinity—see comments below) for different fixed hydrolysis times. This is in line with previous reports in the literature that relate cellulose crystallinity with its proneness to react.^17,34,35^ Nevertheless, the correlation shown in Figure 5 does not intend to neglect the relevance of other factors that have to necessarily have an influence on the reactivity of the pretreated samples, particularly in the high crystallinity domain. For example, for the Avicel samples pretreated with the eutectics of [P_4 4 4 4_][OAc] + [P_4 4 4 4_]Cl and [P_4 4 4 4_][OAc] + [P_4 4 4 4_]Br, a certain improvement of the rate of enzymatic hydrolysis can be observed with respect to untreated Avicel (Figure 4, plots a and b), despite the inability of the corresponding pretreatments to induce any crystallinity reduction (Table 1). In a similar vein, the hydrolyses of the samples pretreated with the [P_4 4 4 4_][OAc] + ethanol mixtures proceeded somewhat slower than that of the sample pretreated with pure [P_4 4 4 4_][OAc], in spite of a practically equivalent *CI* value. For this sample pretreated with pure [P_4 4 4 4_][OAc], it can be finally noted that, although the reduction in *CI* was rather moderate (from 51% down to 46%), the conversion percentage in its hydrolysis roughly duplicated that of untreated Avicel for any given time in the range tested.

### Carboxymethylation

CMC is one of the most attractive cellulose-derived products due to its numerous fields of application: oil recovery drilling fluids, adhesives, coatings, detergents, and importantly the food, cosmetics, and pharma sectors.^36,37^ Such versatility is due to the thickening and gelling properties imparted by CMC salts to aqueous media, allowing rheology control at low cost and with little toxicity and remarkable biodegradability. The process of production of Na-CMC involves a first activation treatment of cellulose under strongly alkaline conditions with an excess of base (generally NaOH), followed by alkylation with chloroacetate at temperatures typically above 45–50 °C in a heterogeneous regime with the cellulose suspended in a fibrous state in an intermediate-polarity alcohol (most commonly, 2-propanol).^38^ Moreover, excess of base above 0.8 equiv with respect to anhydroglucose units is typical, and hence, post-synthesis neutralization of significant residual [OH]^−^ is required.^37,39^

**Figure 6:**
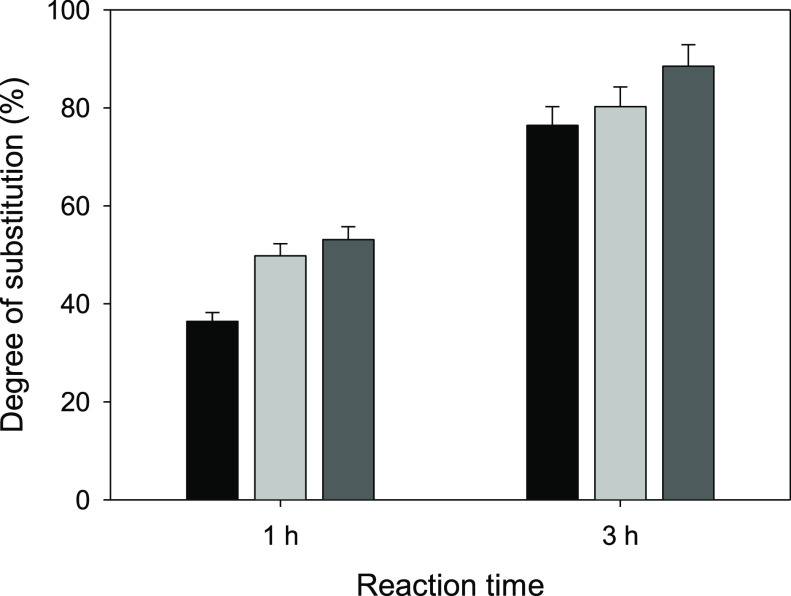
*DS* in the carboxymethylation of untreated Avicel (black columns) and of Avicel samples pretreated with pure [P_4 4 4 4_][OAc] (light gray columns) or with a mixture of [P_4 4 4 4_][OAc] + DMSO with *x*_DMSO_ = 0.20 (dark gray columns) after reaction times of 1 and 3 h. The ATR-FTIR spectra used for the calculation of these *DS* values are shown in Figure S15 in the Supporting Information.

In view of the improved reactivity imparted by the pretreatments explored in this work, a simplified and milder process for the preparation of Na-CMC from Avicel cellulose was investigated, avoiding the alkaline activation stage as well as the excess of base, and using a low reaction temperature (40 °C). The samples pretreated at 70 °C with [P_4 4 4 4_][OAc] (*CI* = 46%) and with the mixture of [P_4 4 4 4_][OAc] + DMSO with *x*_DMSO_ = 0.20 (*CI* = 20%) were particularly selected for this study, together with untreated Avicel (*CI* = 51%) for comparison. After a typical reaction time of 3 h, *DS* correlated inversely with *CI* of the feedstock, as it could be expected. The results are shown in Figure 6. Although even for the untreated Avicel its *DS* = 0.76 falls in the range of 0.7–1.2 which is industrially accepted for the use of CMC as a thickening or gelling agent, it is worth noting that only 12% of the initial [OH]^−^ did not result in carboxymethylation for the sample pretreated with [P_4 4 4 4_][OAc] + DMSO (*DS* = 0.88). This is an important fact since free [OH]^−^ is known to promote the unproductive side reaction involving chloroacetate conversion into glycolate, and if unreacted, hydroxide must be neutralized prior to the isolation of CMC.^37,39^ This *DS* value of 0.88 obtained for Avicel in just 3 h at 40 °C without excess of NaOH is comparable, for example, to that of 0.89 reported by Klug for the preparation of CMC from chemically purified cotton linters under clearly harsher conditions (representative of the industrial state-of-the-art): preactivation with NaOH in a 4 h reaction at 55 °C with NaOH excess of 103 mol % with respect to the anhydroglucose units.^39^

Shortening the reaction time to 1 h, the results (see also Figure 6) show a more marked difference between the *DS* achieved with the untreated Avicel (0.36) and those of the pretreated samples (≥0.50). A *DS* = 0.53 for the sample pretreated with [P_4 4 4 4_][OAc] + DMSO represents, in fact, an improvement of 47% in the substitution efficiency with respect to the untreated Avicel. Interestingly, the *DS* values achieved in this short 1 h reaction time for the pretreated Avicel samples, and especially for those of lower crystallinity pretreated with the mixture of [P_4 4 4 4_][OAc] + DMSO, lie in the range of 0.5–0.6 at which Na-CMC becomes water-soluble, depending on substitution distribution;^37,38^ thus, they might already exhibit favorable gelling properties.

## Conclusions

The ionic liquid [P_4 4 4 4_][OAc], and even more pronouncedly its mixtures with DMSO (*x*_DMSO_ = 0.20 or 0.40), are capable of reducing the crystallinity of the popular commercial microcrystalline cellulose-type Avicel PH-101 by means of a non-dissolving pretreatment under mild conditions (atmospheric pressure and 70 °C—or even a lower temperature in the case of the mixtures of [P_4 4 4 4_][OAc] + DMSO), with easy recovery of the pretreated cellulose and of the pretreatment fluid. This reduction in crystallinity leads to a remarkable improvement of the Avicel reactivity, exemplified herein through a faster rate of enzymatic hydrolysis, as well as through an efficient carboxymethylation reaction carried out under simpler and milder conditions than the benchmark process for industrial production of CMC.

The use of mixtures of [P_4 4 4 4_][OAc] + ethanol (*x*_ethanol_ = 0.20 or 0.40) as pretreatment fluids causes similar reductions in the crystallinity of the Avicel cellulose than the pure [P_4 4 4 4_][OAc], although the improvement in reactivity, evaluated with the kinetics of the enzymatic hydrolysis of the pretreated samples, is somewhat lower. The eutectic compositions of the binary systems constituted by [P_4 4 4 4_][OAc] and its homologous halides [P_4 4 4 4_]Cl and [P_4 4 4 4_]Br were ineffective in pretreating Avicel adequately, with no reduction in the *CI*, and with just a minor improvement over untreated Avicel in the enzymatic hydrolysis kinetics.

Interestingly, the *DP* and the thermal stability of Avicel remain essentially unaffected after the pretreatment with any of the fluids investigated in this work.

In future work, the pretreatment of lignocellulosic biomass is envisioned for improved accessibility and subsequent facilitated reactivity (e.g., toward enzymatic hydrolysis) of its cellulosic fraction directly within the lignocellulosic matrix.

## Supporting Information Available

NMR spectra of tetra(*n*-butyl)phosphonium salts, UV–vis spectrophotometry calibration lines, calibration of ATR-FTIR spectroscopy method for the determination of *DS* in CMC samples, density and viscosity of pretreatment liquids, numerical values of solid–liquid equilibrium data, and ATR-FTIR spectra of the Na-CMC samples obtained in the carboxymethylation experiments (PDF)
