# Supplementary material for: Liquid Systems Based on Tetra(n-butyl)phosphonium Acetate for the Non-dissolving Pretreatment of a Microcrystalline Cellulose (Avicel PH-101)
Source: Biomacromolecules. 2022 Apr 26;23(5):1970–80. doi: 10.1021/acs.biomac.1c01683 (PMC9092347; doi:10.1021/acs.biomac.1c01683)
Supplement: Supplementary file 1 — bm1c01683_si_001.pdf [file bm1c01683_si_001.pdf]

SUPPORTING INFORMATION

Liquid Systems Based on Tetra(*n*-butyl)phosphonium Acetate for the Non-dissolving Pretreatment of a Microcrystalline Cellulose (Avicel PH-101)

Carlos A. Pena,[§] Alberto V. Puga,[†] Andreas Metlen,[‡] Ana Soto,[§] and Héctor Rodríguez[§,*]

[§] *CRETUS, Department of Chemical Engineering, Universidade de Santiago de Compostela, E-15782, Santiago de Compostela, Spain*

[†] *Departament d'Enginyeria Química, Universitat Rovira i Virgili, Avinguda dels Països Catalans 26, 43007 Tarragona, Spain*

[‡] *AMT1 – Translations & Chemistry, Schuurblok 11, 2910 Essen, Antwerp, Belgium*

* Corresponding author. E-mail: hector.rodriguez@usc.es. Phone: +34 881816804.

Contents:

- ^{1}H and ^{13}C NMR spectra of the purified tetra(*n*-butyl)phosphonium salts S2
- UV-vis spectrophotometric calibration lines for the tetra(*n*-butyl)phosphonium salts in water S5
- Calibration of the ATR-FTIR spectroscopy method for the determination of the degree of substitution (*DS*) of carboxymethylated cellulose samples S7
- Density and viscosity of the pretreatment liquids S12
- Numerical values of the solid-liquid equilibrium data for the binary systems $[P_{4\,4\,4\,4}][OAc]$ + ethanol and $[P_{4\,4\,4\,4}][OAc]$ + DMSO S15
- ATR-FTIR spectra of the Na-CMC samples obtained in the carboxymethylation experiments S16

^{1}H and ^{13}C NMR spectra of the purified tetra(*n*-butyl)phosphonium salts

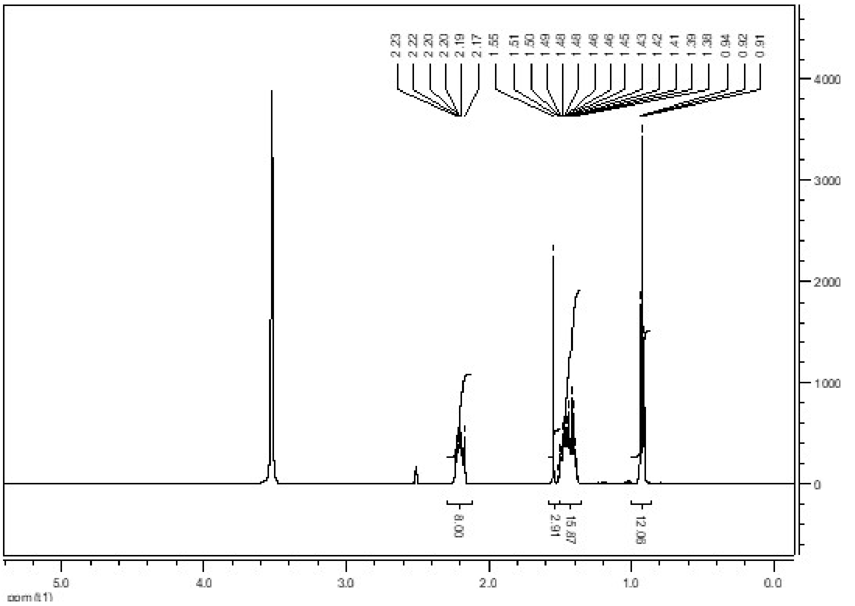

Figure S1. ^{1}H NMR spectrum of [$P_{4\,4\,4\,4}$][OAc], δ_H (DMSO-d_6, 300 MHz): 0.92 (t, J = 7.1 Hz, 12H, 4 × $P(CH_2)_3C\underline{H}_3$), 1.34-1.52 (unresolved, 16H, 4 × $PCH_2(C\underline{H}_2)_2$), 1.55 (s, 3H, CH_3COO), 2.12-2.25 (unresolved, 8H, 4 × PCH_2). The peaks at 2.5 ppm and 3.6 ppm correspond, respectively, to the residual proton signal of the perdeuterated solvent and to traces of water present in the mixture (solvent + sample).

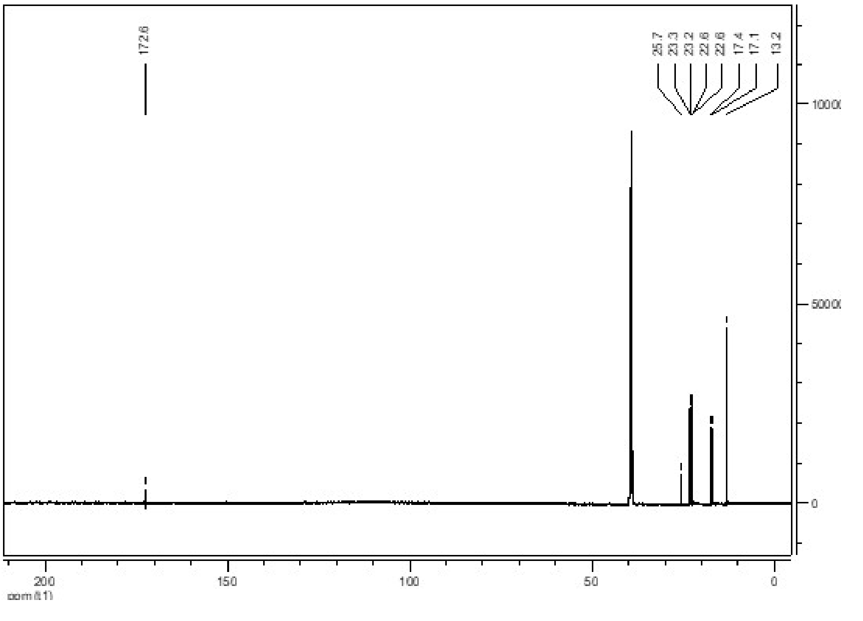

Figure S2. ^{13}C NMR spectrum of [$P_{4\,4\,4\,4}$][OAc], δ_C (DMSO-d_6, 75.4 MHz): 13.2 (s, 4 × $P(CH_2)_3\underline{C}H_3$), 17.3 (d, $J_{C\text{-}P}$ = 48 Hz, 4 × PCH_2), 22.6 (d, J = 4 Hz, 4 × $P(CH_2)_2\underline{C}H_2$), 23.3 (d, J = 16 Hz, 4 × $PCH_2\underline{C}H_2$), 25.7 (s, $\underline{C}H_3COO$), 172.6 (s, $CH_3\underline{C}OO$). The peak at 39.4 ppm corresponds to the perdeuterated solvent.

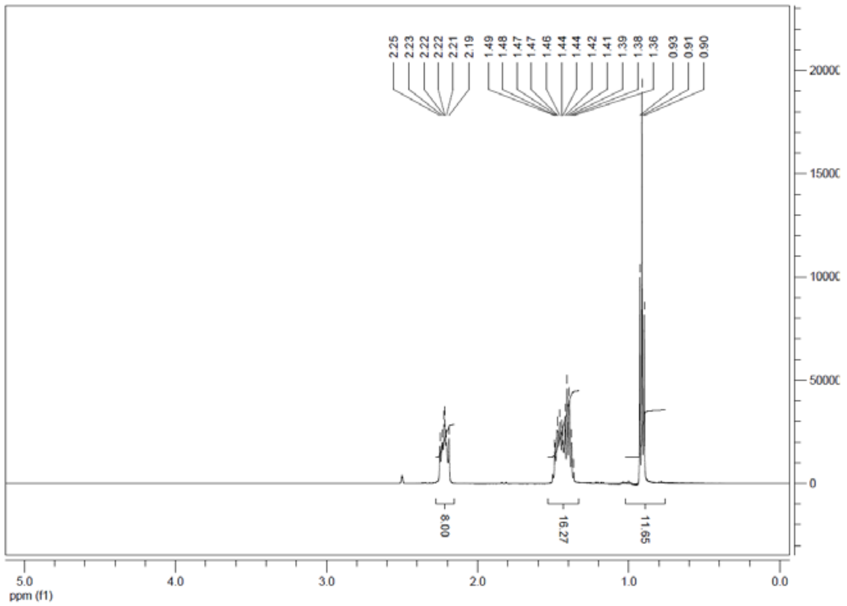

Figure S3. ^{1}H NMR spectrum of $[P_{4\,4\,4\,4}]Cl$, δ_H (DMSO-d$_6$, 300 MHz): 0.91 (t, J = 7.1 Hz, 12H, 4 × $P(CH_2)_3C\underline{H}_3$), 1.34-1.51 (unresolved, 16H, 4 × $PCH_2(C\underline{H}_2)_2$), 2.18-2.26 (unresolved, 8H, 4 × PCH_2). The peak at 2.5 ppm corresponds to the residual proton signal of the perdeuterated solvent.

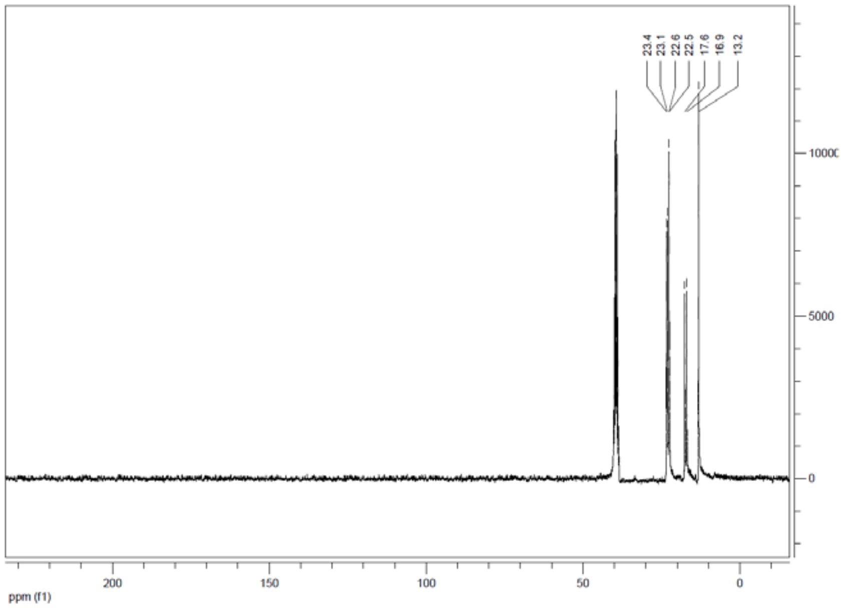

Figure S4. ^{13}C NMR spectrum of $[P_{4\,4\,4\,4}]Cl$, δ_C (DMSO-d$_6$, 75.4 MHz): 13.2 (s, 4 × $P(CH_2)_3\underline{C}H_3$), 17.3 (d, $J_{C\text{-}P}$ = 48 Hz, 4 × PCH_2), 22.6 (d, J = 4 Hz, 4 × $P(CH_2)_2\underline{C}H_2$), 23.2 (d, J = 16 Hz, 4 × $PCH_2\underline{C}H_2$). The peak at 39.4 ppm corresponds to the perdeuterated solvent.

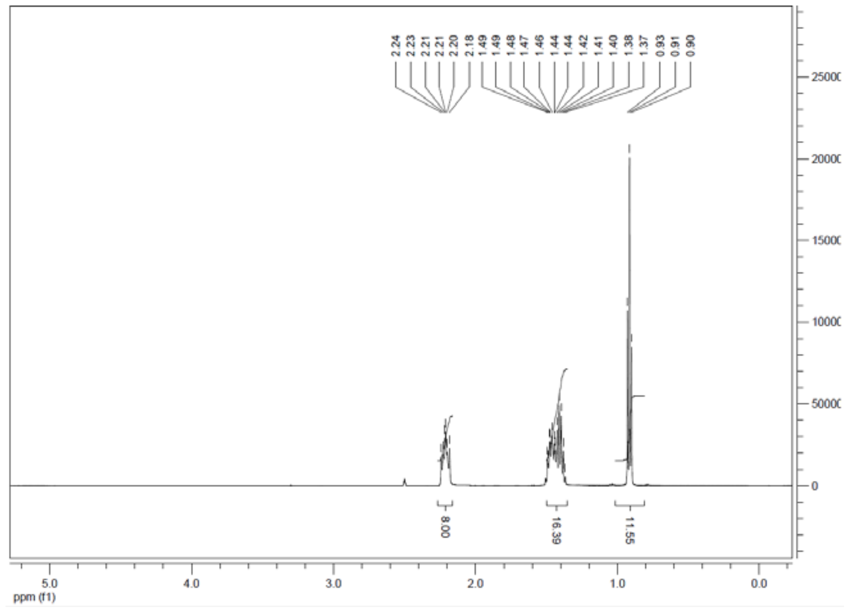

Figure S5. ^{1}H NMR spectrum of $[P_{4\,4\,4\,4}]Br$, δ_H (DMSO-d_6, 300 MHz): 0.91 (t, J = 7.1 Hz, 12H, 4 × $P(CH_2)_3C\underline{H}_3$), 1.31-1.55 (unresolved, 16H, 4 × $PCH_2(C\underline{H}_2)_2$), 2.09-2.28 (unresolved, 8H, 4 × PCH_2). The peak at 2.5 ppm corresponds to the residual proton signal of the perdeuterated solvent.

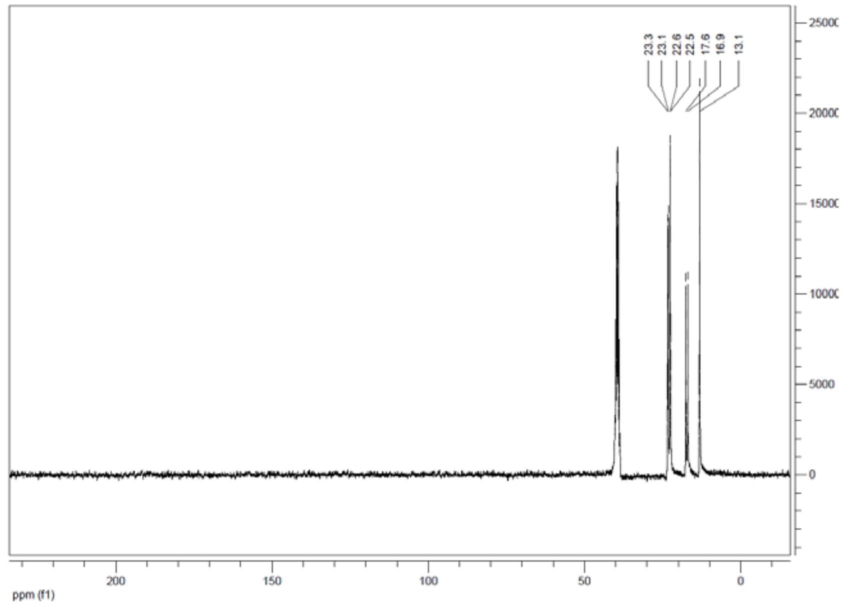

Figure S6. ^{13}C NMR spectrum of $[P_{4\,4\,4\,4}]Br$, δ_C (DMSO-d_6, 75.4 MHz): 13.1 (s, 4 × $P(CH_2)_3\underline{C}H_3$), 17.3 (d, $J_{C\text{-}P}$ = 48 Hz, 4 × PCH_2), 22.6 (d, J = 4 Hz, 4 × $P(CH_2)_2\underline{C}H_2$), 23.2 (d, J = 16 Hz, 4 × $PCH_2\underline{C}H_2$). The peak at 39.4 ppm corresponds to the perdeuterated solvent.

UV-vis spectrophotometric calibration line for the tetra(*n*-butyl)phosphonium salts in water

Table S1. Experimentally measured UV-vis absorbance, at 195 nm, of aqueous solutions of $[P_{4\,4\,4\,4}][OAc]$.

Concentration of $[P_{4\,4\,4\,4}][OAc]$ (mg/L)	Absorbance at 195 nm (a. u.)
10	0.037
100	0.116
250	0.399
500	0.711
750	0.952
1000	1.235

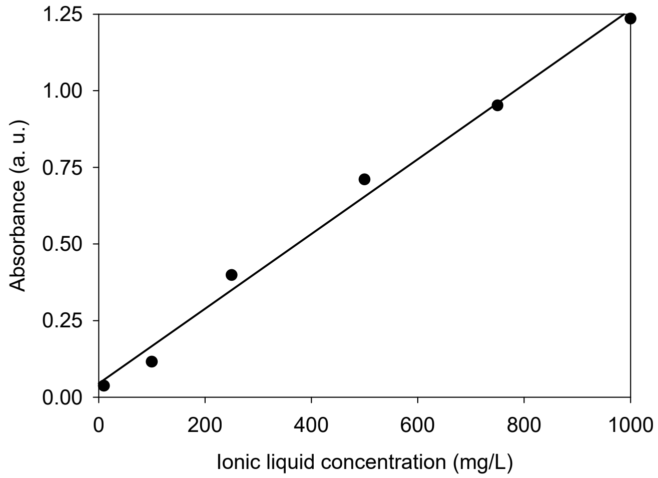

Figure S7. Calibration line for the quantification of ionic liquid in the aqueous washings of cellulose after pretreatment with $[P_{4\,4\,4\,4}][OAc]$ or with its mixtures with ethanol or DMSO. Parameters of the linear fit: slope = 1.22×10^{-3} L/mg; intercept = 0.0435.

Table S2. Experimentally measured UV-vis absorbance, at 195 nm, of aqueous solutions of the eutectic mixture of $[P_{4\,4\,4\,4}][OAc]$ and $[P_{4\,4\,4\,4}]Cl$ ($x_{[P4444][OAc]}$ = 0.50).

Concentration of the eutectic mixture of $[P_{4\,4\,4\,4}][OAc]$ + $[P_{4\,4\,4\,4}]Cl$ (mg/L)	Absorbance at 195 nm (a. u.)
10	0.035
80	0.055
200	0.129
500	0.252
750	0.342

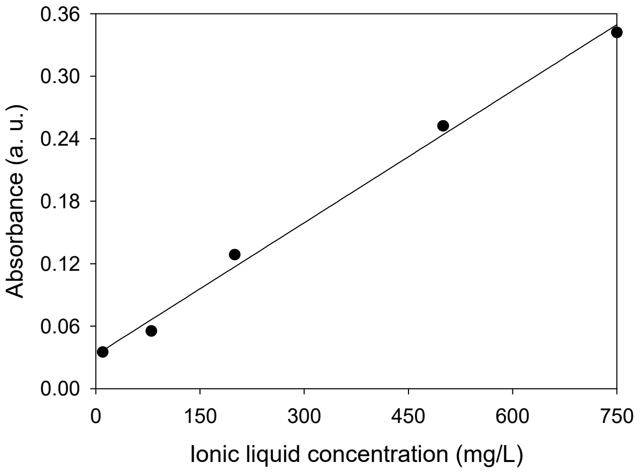

Figure S8. Calibration line for the quantification of ionic liquid in the aqueous washings of cellulose after pretreatment with the eutectic mixture of $[P_{4\,4\,4\,4}][OAc]$ + $[P_{4\,4\,4\,4}]Cl$. Parameters of the linear fit: slope = 4.23×10^{-4} L/mg; intercept = 0.0324.

Table S3. Experimentally measured UV-vis absorbance, at 210 nm, of aqueous solutions of the eutectic mixture of $[P_{4\,4\,4\,4}][OAc]$ + $[P_{4\,4\,4\,4}]Br$ ($x_{[P4444][OAc]}$ = 0.70).

Concentration of the eutectic mixture of $[P_{4\,4\,4\,4}][OAc]$ + $[P_{4\,4\,4\,4}]Br$ (mg/L)	Absorbance at 195 nm (a. u.)
40	0.055
150	0.192
353	0.435
705	0.851

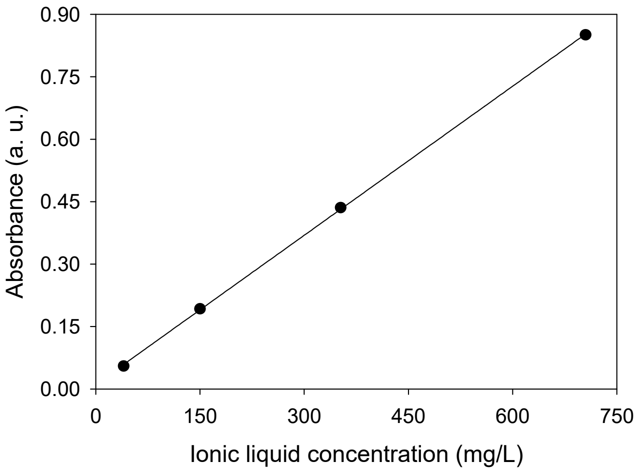

Figure S9. Calibration line for the quantification of the ionic liquid in the aqueous washings of cellulose after pretreatment with the eutectic mixture of $[P_{4\,4\,4\,4}][OAc]$ + $[P_{4\,4\,4\,4}]Br$. Parameters of the linear fit: slope = 1.19×10^{-3} L/mg; intercept = 0.0111.

Calibration of the ATR-FTIR spectroscopy method for the determination of the degree of substitution (*DS*) of carboxymethylated cellulose samples

To calibrate the method based on the carboxylate vs. methylenic/methinic absorbance ratio (R_{CM}) measured by ATR-FTIR spectroscopy, the *DS* for a series of carboxymethylcellulose samples in sufficiently large amounts were determined by acid-base back-titration, and then correlated to the R_{CM} values obtained by ATR-FTIR. Such carboxymethylcellulose comprised a commercial one (sodium carboxymethylcellulose, M_w ~ 250,000, degree of substitution 1.2, Sigma-Aldrich) and a range of samples prepared by standard procedures varying solvent composition (2-propanol/water ratios) and/or reaction time – See Table S4 and paragraphs below. This covered a wide *DS* range (0–1.2). The resulting linear regression correlating *DS* and R_{CM}, shown in Figure S10, presents a satisfactory coefficient of correlation: $R^2 = 0.981$.

Table S4. Carboxymethylcellulose samples used for the calibration of the ATR-FTIR method *vs.* titration for the determination of *DS*.

Entry	2-Propanol/water ratio (*v*/*v*)	Time (h)	R_{CM} (ATR-FTIR)	*DS* (titration)
1[a]	-	-	5.14	1.18
2[b]	10.8	3	3.76	0.76
3[b]	10.8	3	3.91	0.81
4[b]	2	3	1.59	0.18
5[b]	2	1	0.93	0.16
6[b]	2	0.5	0.93	0.14
7[b]	1	3	0.54	0.09
8[c]	2	3	1.45	0.23
9[c]	2	0.5	1.11	0.18
10[b]	0	3	0	0

[a] From commercial sodium carboxymethylcellulose (M_w ~ 250,000, degree of substitution 1.2, Sigma-Aldrich) after acidification.
[b] From Avicel after carboxymethylation and subsequent acidification.
[c] From Avicel after pretreatment with $[P_{4444}][OAc]$ + DMSO ($x_{DMSO} = 0.20$), carboxymethylation and subsequent acidification.

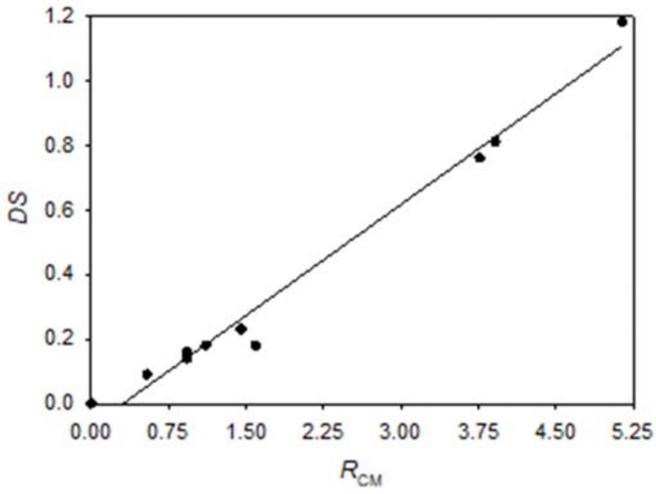

Figure S10. Correlation between *DS* determined by acid-bas back-titration and the carboxylate vs. methylenic/methinic absorbance ratio measured by ATR-FTIR (R_{CM}) for the samples listed in Table S4. Parameters of the linear fit: slope = 0.229; intercept = -0.072.

Carboxymethylation. The carboxymethylation of cellulose samples in sufficiently large amount for titration analyses was performed by a procedure adapted from Pushpamalar et al. (2006). In a typical carboxymethylation, a cellulose sample (0.500 g) was weighted in a 25 mL round-bottomed flask, and it was suspended in 2-propanol (Scharlau ACS Basic, ≥99.5 %, 10 mL). A volume of 1.0 mL of a 20 % w/v aqueous solution of sodium hydroxide (Sigma-Aldrich, ≥98 %) was then added dropwise under magnetic stirring, and the resulting suspension further stirred at 500 rpm for 1 h at room temperature. The flask was then immersed in an oil bath preheated at 40 °C and the suspension stirred at 700 rpm for 2 min. Sodium chloroacetate (Sigma-Aldrich, for synthesis, 0.600 g) was added and the suspension stirred at 700 rpm for the desired time (0.5, 1 or 3 h). After reaction, the flask was taken out of the bath, allowed to cool down to room temperature and the solid separated by filtration through a sintered glass funnel (pore size: 3), washed with 2-propanol (3 × 3 mL) and air-dried by suction. The collected solid was suspended in methanol (Merck, ACS reagent, ≥ 99.9%, 30 mL) and the stirred mixture neutralised until pH ≈ 6, as measured using pH indicator paper, by adding the required amount of glacial acetic acid (Scharlau, extrapure). The solid was separated by filtration on a sintered glass funnel (pore size: 3), washed with methanol (5 × 3 mL) and dried under an air stream by suction. The final sodium carboxymethylcellulose (Na-CMC) samples had a white colour and their texture ranged from powdery solids to slightly sticky flakes depending on their degrees of substitution.

Acidification. Conversion of Na-CMC samples into their acid form was carried out by adapting procedures described in the literature (Eyler et al., 1947; Pushpamalar et al., 2006). The procedure varied slightly depending on whether the CMC was commercial or had been synthesised from cellulose in our laboratories.

In a typical acidification procedure for a sample prepared from cellulose, Na-CMC (0.500 g) was dispersed in ethanol (Scharlau Pharmpur®, 96 % v/v, 10 mL) in a 25 mL round-bottom flask connected to a condenser. The suspension was heated in an oil bath at 90 °C and stirred at 1000 rpm until boiling. Then, an aqueous solution (2.0 M, 1.7 mL) of nitric acid (J. T. Baker, 65 %) was added dropwise and the boiling mixture stirred for 5 min at 1000 rpm, and then for further 15 min outside the oil bath while cooling down to room temperature. The solid was separated by filtration through a sintered glass funnel (pore size: 3), washed with ethanol (5 ×4 mL), further washed with an ethanol/water mixture (80:20 v/v, ca. 50 mL) until the pH of the liquor was >6, as detected by using pH indicator paper, and air-dried by suction. The resulting solid was identified as the acid form of carboxymethylcellulose (H-CMC) based on ATR-FTIR spectroscopy (see Figure S11). Before titration, the H-CMC was placed in a Petri dish and dried in an oven at 105 °C for 1 h or until constant weight. The resulting weight was taken as its dry weight for *DS* quantification.

Acidification of commercial Na-CMC was performed under a more diluted and less aqueous regime to avoid gel formation, a phenomenon which seriously hinders solid recovery. In a typical procedure, Na-CMC (1.00 g) was dispersed in methanol (Merck, ACS Reagent, ≥99.9 %, 200 mL) in a 500 mL round-bottomed flask connected to a condenser. Nitric acid (J. T. Baker, 65 %, 2.0 mL) was added dropwise, the resulting suspension heated in an oil bath at 80 °C and stirred at 1000 rpm until boiling, then stirred for 5 min at 1000 rpm while boiling, and taken out of the oil bath while cooling down to room temperature under stirring. The solid was separated by filtration through a sintered glass funnel (pore size: 3), washed with methanol (5 × 10 mL), further washed with a methanol/water mixture (80:20 v/v, *ca*. 200 mL) until the pH of the liquor was >6, as detected by using pH indicator paper, and air-dried by suction. As above, the resulting solid was identified as the acid form of carboxymethylcellulose (H-CMC) based on ATR-FTIR spectroscopy. Before titration, the H-CMC was placed in a Petri dish and dried in an oven at 105 °C until constant weight. The resulting weight was taken as its dry weight for *DS* quantification.

Titration. The *DS* of CMC samples was accurately determined by an acid-base back-titration method in aqueous solution consisting of a prior basification of H-CMC samples using a sodium

hydroxide solution, and the titration of the remaining free $[OH]^-$ by a hydrochloric acid solution. The difference between such remaining $[OH]^-$ and that of a blank titration of an equal amount of sodium hydroxide (without H-CMC) is used to determine the amount of moles of acid in the CMC sample.

In a typical back-titration procedure, an accurately weighted sample of one of the prepared H-CMC materials (*ca.* 0.3000 g, corrected to only account for its dry weight, as described above) was dispersed in deionized water (60 mL) in a conical flask and heated to boiling on a hot plate. At this stage, an aqueous sodium hydroxide solution (0.2 M, 20.00 ± 0.03 mL) was added, and the mixture shaken. Samples of high *DS* (>0.6) readily dissolved after a few seconds, whereas the solid remained suspended for samples of lower *DS*. In either case, the aqueous mixtures were titrated with an aqueous solution of hydrochloric acid (Panreac, for analysis, 37% *w*/*w*), standardised by conventional methods (0.0997 M) by using phenolphthalein as an indicator. The blank titrations were performed by a similar procedure, yet in the absence of H-CMC.

The molar concentration of carboxymethyl groups of the H-CMC (C_{CM}, expressed in $mol_{acid}/g_{H\text{-}CMC}$) was calculated according to:

$$C_{CM} = \frac{\left(v_{HCl,blank} - v_{HCl,sample}\right) \times C_{HCl}}{m_{sample}}$$

where $v_{HCl,blank}$ and $v_{HCl,sample}$ are the volumes (in litres) of the HCl solution employed for the blank and sample titrations, C_{HCl} is the concentration of the standardised HCl solution (0.0997 M), and m_{sample} is the mass of dry H-CMC. The determination of *DS* was then done by considering that:

$$DS = \frac{n_{CM}}{n_{AG}}$$

where n_{CM} and n_{AG} are the moles of carboxymethyl groups and anhydroglucose units, respectively, in an H-CMC sample (assumed to be equal to those of the parent Na-CMC sample). Operating with the above equations, we can get:

$$DS = \frac{MW_{AG} \times C_{CM}}{1 - MW_{CM} \times C_{CM}}$$

where MW_{AG} and MW_{CM} are the molar weights of anhydroglucose ($C_6H_{10}O_5$) and carboxymethyl (CH_2COO) units, respectively (Eyler et al., 1947). The *DS* values thereby obtained are listed in Table S4.

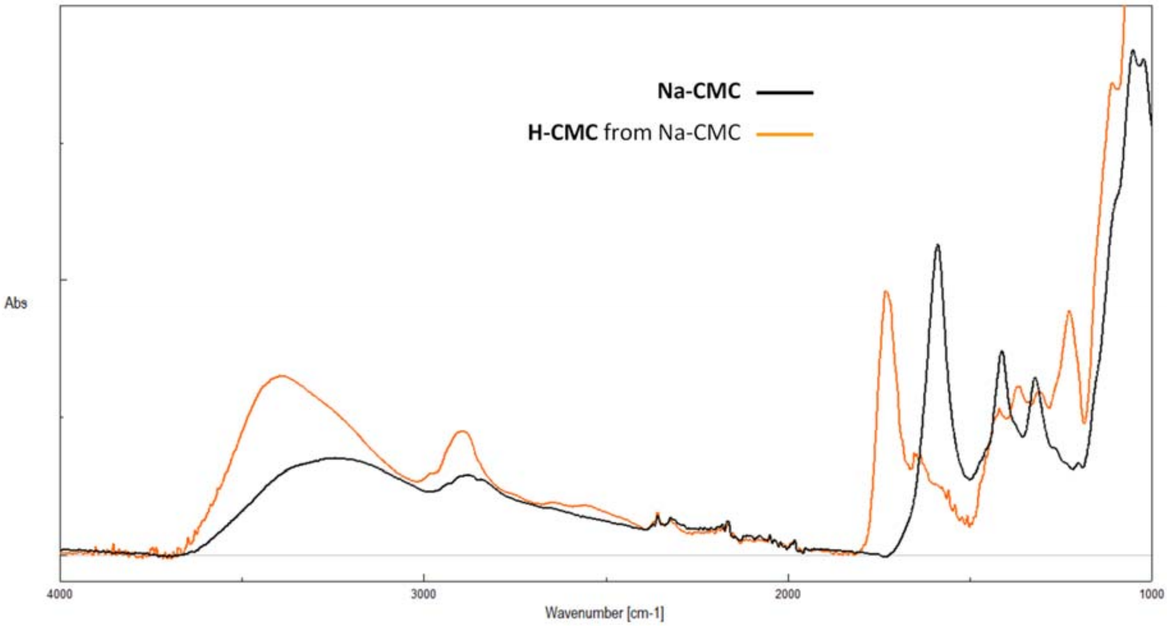

Figure S11. ATR-FTIR spectra of sodium carboxymethylcellulose (Na-CMC) prepared from Avicel after 3 h reaction time, as for Entry 3 in Table S4, as compared to its acid form (H-CMC) prepared for determination of *DS* by titration. The effective conversion from sodium to protonic form is confirmed by the disappearance of the asymmetric carboxylate stretching band (*ca*. 1590 cm^{-1}) and the appearance of the carboxylic (C=O) stretching signal (*ca*. 1730 cm^{-1}).

References:

Eyler, R. W., Klug, E. D., & Diephuis, F. (1947). Determination of Degree of Substitution of Sodium Carboxymethylcellulose. *Analytical Chemistry*, *19*, 24-27. https://doi.org/10.1021/ac60001a007

Pushpamalar, V., Langford, S. J., Ahmad, M., & Lim, Y. Y. (2006). Optimization of reaction conditions for preparing carboxymethyl cellulose from sago waste. *Carbohydrate Polymers*, *64*, 312-318. https://doi.org/10.1016/j.carbpol.2005.12.003

Density and viscosity of the pretreatment liquids

Table S5. Experimentally measured density (ρ) and viscosity (η) of the pretreatments fluids based on the combination of $[P_{4\,4\,4\,4}][OAc]$ with $[P_{4\,4\,4\,4}]Cl$, $[P_{4\,4\,4\,4}]Br$, ethanol, or DMSO, at different temperatures *T*.

T (°C)	ρ (g/cm^3)	η (mPa·s)	T (°C)	ρ (g/cm^3)	η (mPa·s)
$[P_{4\,4\,4\,4}][OAc]$ + $[P_{4\,4\,4\,4}]Cl$, $x_{[P4444][OAc]}$ = 0.50			$[P_{4\,4\,4\,4}][OAc]$ + $[P_{4\,4\,4\,4}]Br$, $x_{[P4444][OAc]}$ = 0.70		
40.00	0.93018	799.4	40.00	0.96358	412.5
45.00	0.92734	550.8	45.00	0.96062	297.0
50.00	0.92449	393.7	50.00	0.95766	217.8
55.00	0.92166	286.9	55.00	0.95470	163.3
60.00	0.91882	211.6	60.00	0.95174	124.3
65.00	0.91599	157.0	65.00	0.94877	106.3
70.00	0.91315	121.1	70.00	0.94583	85.20
75.00	0.91031	93.92	75.00	0.94289	69.35
80.00	0.90748	74.37	80.00	0.93994	54.82
$[P_{4\,4\,4\,4}][OAc]$ + ethanol, $x_{ethanol}$ = 0.20			$[P_{4\,4\,4\,4}][OAc]$ + ethanol, $x_{ethanol}$ = 0.40		
40.00	0.92125	133.2	40.00	0.91316	71.05
45.00	0.91826	100.9	45.00	0.91011	55.75
50.00	0.91527	77.94	50.00	0.90707	44.57
55.00	0.91229	61.19	55.00	0.90404	35.97
60.00	0.90932	48.77	60.00	0.90100	29.50
65.00	0.90635	39.57	65.00	0.89797	24.56
70.00	0.90338	32.38	70.00	0.89494	20.63
75.00	0.90042	31.52	75.00	0.89192	17.58
80.00	0.89747	27.29	80.00	0.88890	14.91
$[P_{4\,4\,4\,4}][OAc]$ + DMSO, x_{DMSO} = 0.20			$[P_{4\,4\,4\,4}][OAc]$ + ethanol, x_{DMSO} = 0.40		
40.00	0.93356	115.2	40.00	0.94390	68.53
45.00	0.93055	91.01	45.00	0.94078	53.42
50.00	0.92755	71.24	50.00	0.93768	42.35
55.00	0.92455	55.97	55.00	0.93458	34.06
60.00	0.92156	45.03	60.00	0.93148	27.76
65.00	0.91857	36.52	65.00	0.92840	22.91
70.00	0.91559	29.87	70.00	0.92531	19.49
75.00	0.91261	25.00	75.00	0.92223	16.70
80.00	0.90962	21.10	80.00	0.91914	14.20

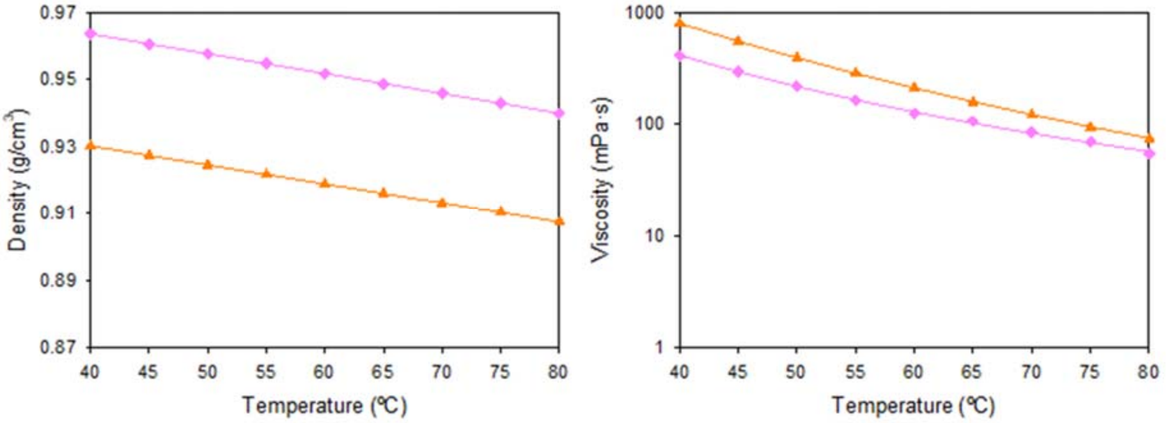

Figure S12. Density (plot on the left) and viscosity (plot on the right) for the eutectic compositions of the mixtures $[P_{4\,4\,4\,4}][OAc]$ + $[P_{4\,4\,4\,4}]Cl$ ($x_{[P4444][OAc]}$ = 0.50) (orange) and $[P_{4\,4\,4\,4}][OAc]$ + $[P_{4\,4\,4\,4}]Br$ ($x_{[P4444][OAc]}$ = 0.70) (pink). Solid lines correspond to the fits provided by the equations and parameters in Table 1 of the main manuscript.

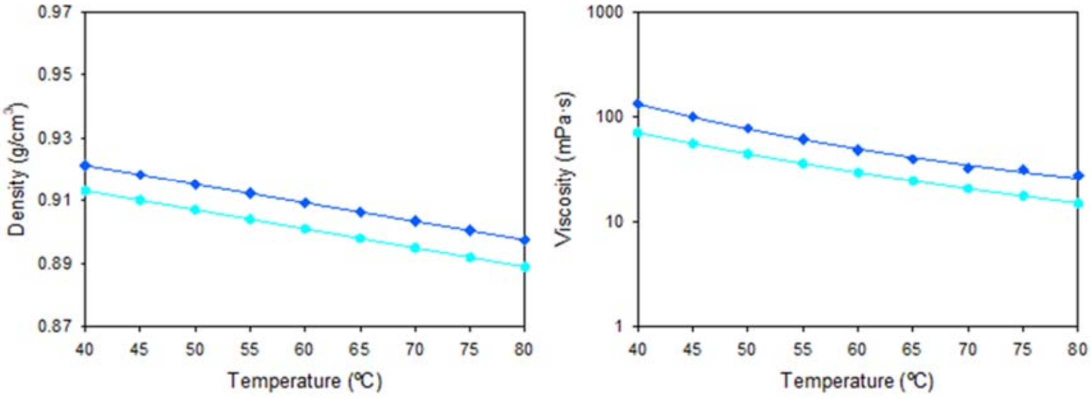

Figure S13. Density (plot on the left) and viscosity (plot on the right) for mixtures $[P_{4\,4\,4\,4}][OAc]$ + ethanol, with $x_{ethanol}$ = 0.20 (dark blue) and $x_{ethanol}$ = 0.40 (light blue). Solid lines correspond to the fits provided by the equations and parameters in Table 1 of the main manuscript.

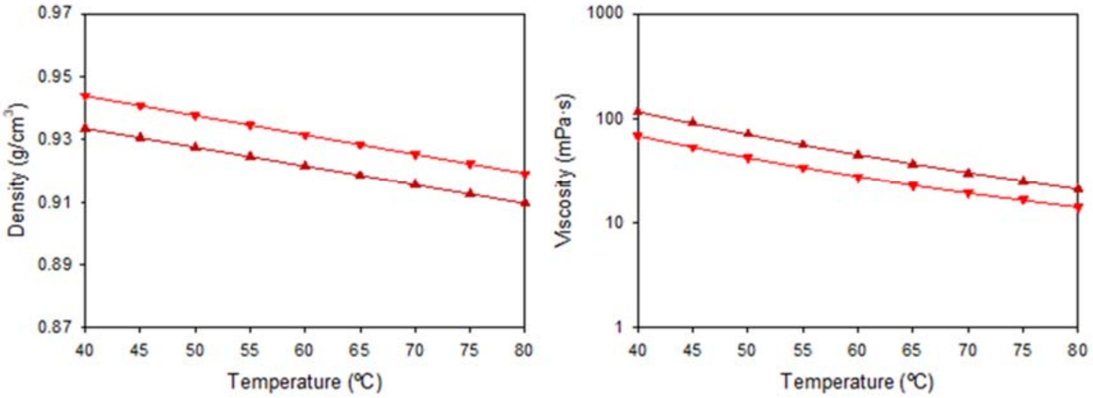

Figure S14. Density (plot on the left) and viscosity (plot on the right) for mixtures $[P_{4\,4\,4\,4}][OAc]$ + DMSO, with $x_{DMSO} = 0.20$ (dark red) and $x_{DMSO} = 0.40$ (light red). Solid lines correspond to the fits provided by the equations and parameters in Table 1 of the main manuscript.

Table S6. Parameters for the correlation of density (with a polynomial equation: $\rho = a + b \cdot T + c \cdot T^2$) and viscosity (with the Vogel-Fulcher-Tammann equation: $\eta = A \cdot T^{1/2} \cdot \exp(k/(T\text{-}T_0))$, as a function of absolute temperature T (in K), in the temperature range from 313.15 K to 353.15 K, for the pretreatment liquids based on the combination of $[P_{4\,4\,4\,4}][OAc]$ with $[P_{4\,4\,4\,4}]Cl$, $[P_{4\,4\,4\,4}]Br$, ethanol, or DMSO [a,b].

Pretreatment liquid	a	$b \times 10^4$	$c \times 10^8$	$A \times 10^4$	k	T_0
$[P_{4\,4\,4\,4}][OAc]$ + $[P_{4\,4\,4\,4}]Cl$, $x_{[P4\,4\,4\,4][OAc]} = 0.50$	0.95287	-5.6751	-	8.325	1524	173.3
$[P_{4\,4\,4\,4}][OAc]$ + $[P_{4\,4\,4\,4}]Br$, $x_{[P4\,4\,4\,4][OAc]} = 0.70$	0.98721	-5.9114	-	266.1	631.3	220.0
$[P_{4\,4\,4\,4}][OAc]$ + ethanol, $x_{ethanol} = 0.20$	0.94535	-6.0659	10.099	411.4	427.1	231.2
$[P_{4\,4\,4\,4}][OAc]$ + ethanol, $x_{ethanol} = 0.40$	0.93765	-6.1516	7.1645	28.76	1009	173.8
$[P_{4\,4\,4\,4}][OAc]$ + DMSO, $x_{DMSO} = 0.20$	0.95747	-5.9827	-	8.352	1471	149.2
$[P_{4\,4\,4\,4}][OAc]$ + DMSO, $x_{DMSO} = 0.40$	0.96862	-6.1860	-	45.52	842.8	188.2

[a] The units of the parameters, as expressed in the table, are as follows: a, $g \cdot cm^{-3}$; b, $g \cdot cm^{-3} \cdot K^{-1}$; c, $g \cdot cm^{-3} \cdot K^{-2}$; A, $mPa \cdot s \cdot K^{-0.5}$; k, K; T_0, K.

[b] Parameter c is only displayed in those cases in which the quadratic term of the fit equation was found statistically significant by means of the F test.

Numerical values of the solid-liquid equilibrium data for the binary systems $[P_{4\,4\,4\,4}][OAc]$ + ethanol and $[P_{4\,4\,4\,4}][OAc]$ + DMSO

Table S7. Experimentally determined melting temperatures (T_m) of mixtures of $[P_{4\,4\,4\,4}][OAc]$ and ethanol of different compositions (expressed as mole fractions of ethanol, $x_{ethanol}$), covering the compositional range of the binary system from $x_{ethanol}$ = 0 to $x_{ethanol}$ = 0.70.

$x_{ethanol}$	T_m (°C)
0.000	58
0.104	40
0.200	31
0.296	22
0.395	15
0.497	7
0.600	-4
0.659	-26
0.700	-44

Table S8. Experimentally determined thermal events (T_{exc}: melting temperatures of the excess compounds; T_{eut}: melting temperatures of the eutectic composition) of mixtures of $[P_{4\,4\,4\,4}][OAc]$ and DMSO of different compositions (expressed as mole fractions of DMSO, x_{DMSO}), covering the entire compositional range of the binary system.

x_{DMSO}	T_{exc} (°C)	T_{eut} (°C)
0.000	58	—
0.109	41	-9
0.220	29	-13
0.296	21	-6
0.399	15	-6
0.505	3	-5
0.601	0	-9
0.699	—	-12
0.800	-2	-6
0.900	6	-6
1.000	17	—

ATR-FTIR spectra of the Na-CMC samples obtained in the carboxymethylation experiments

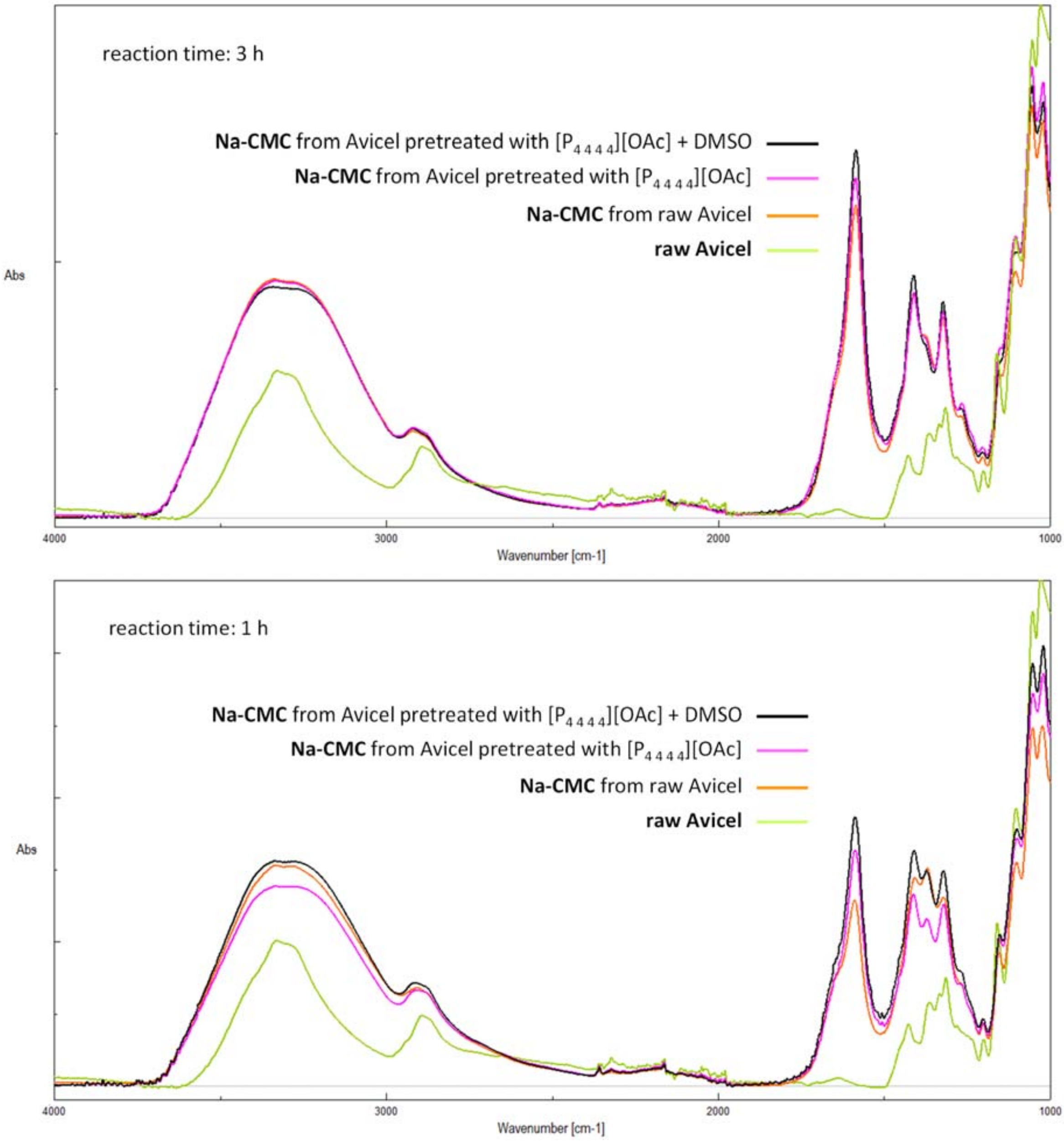

Figure S15. ATR-FTIR spectra of sodium carboxymethyl cellulose (Na-CMC) samples prepared after a reaction time of 3 h (top plot) or 1 h (bottom plot), from untreated Avicel or from Avicel pretreated with pure $[P_{4\,4\,4\,4}][OAc]$ or with a mixture of $[P_{4\,4\,4\,4}][OAc]$ + DMSO (x_{DMSO} = 0.20). The spectrum of raw Avicel is also included for comparison. The extent of carboxymethylation can be observed by the increasing signal at ca. 1590 cm^{-1}. Normalisation of absorbance was performed at the area of ATR artifacts (ca. 2150 cm^{-1}) for a more realistic comparison.
